# Omicron BA.1 breakthrough infection drives cross-variant neutralization and memory B cell formation against conserved epitopes

**DOI:** 10.1126/sciimmunol.abq2427

**Published:** 2022-06-02

**Authors:** Jasmin Quandt, Alexander Muik, Nadine Salisch, Bonny Gaby Lui, Sebastian Lutz, Kimberly Krüger, Ann-Kathrin Wallisch, Petra Adams-Quack, Maren Bacher, Andrew Finlayson, Orkun Ozhelvaci, Isabel Vogler, Katharina Grikscheit, Sebastian Hoehl, Udo Goetsch, Sandra Ciesek, Özlem Türeci, Ugur Sahin

**Affiliations:** ^1^ BioNTech, An der Goldgrube 12, 55131 Mainz, Germany.; ^2^ Institute for Medical Virology, University Hospital, Goethe University Frankfurt, 60596 Frankfurt am Main, Germany.; ^3^ Health Protection Authority, City of Frankfurt, 60313 Frankfurt, Germany.; ^4^ DZIF – German Centre for Infection Research, External Partner Site, 60596 Frankfurt, Germany.; ^5^ HI-TRON – Helmholtz Institute for Translational Oncology Mainz by DKFZ, Obere Zahlbacherstr. 63, 55131 Mainz, Germany.; ^6^ TRON gGmbH – Translational Oncology at the University Medical Center of the Johannes Gutenberg, University Freiligrathstraße 12, 55131 Mainz, Germany.

## Abstract

Omicron is the evolutionarily most distinct SARS-CoV-2 variant of concern (VOC) to date. We report that Omicron BA.1 breakthrough infection in BNT162b2-vaccinated individuals resulted in strong neutralizing activity against Omicron BA.1, BA.2 and previous SARS-CoV-2 VOCs, but not against the Omicron sublineages BA.4 and BA.5. BA.1 breakthrough infection induced a robust recall response, primarily expanding B_MEM_ cells against epitopes shared broadly amongst variants, rather than inducing BA.1-specific B cells. The vaccination-imprinted B_MEM_ cell pool had sufficient plasticity to be remodeled by heterologous SARS-CoV-2 spike glycoprotein exposure. While selective amplification of B_MEM_ cells recognizing shared epitopes allows for effective neutralization of most variants that evade previously established immunity, susceptibility to escape by variants that acquire alterations at hitherto conserved sites may be heightened.

## Teaser

BA.1 infection after BNT162b2 vaccination drives cross-variant neutralization and memory B cell formation against conserved epitopes.

## INTRODUCTION

Containment of the COVID-19 pandemic requires the generation of durable and sufficiently broad immunity to provide protection against current and future variants of SARS-CoV-2. The titer of neutralizing antibodies to SARS-CoV-2, and the binding of antibodies to the spike (S) glycoprotein and its receptor-binding domain (RBD) are considered correlates of protection against infection (*1*, *2*). Currently available vaccines are based on the S glycoprotein of the ancestral Wuhan-Hu-1 strain and induce antibodies with a neutralizing capacity that exceeds the breadth elicited by infection with the Wuhan strain, or with variants of concern (VOCs) (*3*). However, protective titers wane over time (*4*–*7*) and routine booster vaccinations are thought to be needed to trigger recall immunity and maintain efficacy against new VOCs (*8*–*11*).

Long-lived memory B (B_MEM_) cells are the basis for the recall response upon antigen re-encounter either by infection or booster vaccination. They play an important role in the maintenance and evolution of the antiviral antibody response against variants, since low-affinity selection mechanisms during the germinal center reaction and continued hypermutation of B_MEM_ cells over several months following antigen exposure expand the breadth of viral variant recognition (*12*, *13*).

To date, over 1 billion people worldwide have been vaccinated with the mRNA-based COVID-19 vaccine BNT162b2 and have received the primary 2-dose series or further boosters (*14*). Thus, BNT162b2 vaccination is contributing substantially to the pattern of population immunity in many regions of the world.

How vaccine-mediated protective immunity will evolve over time and will be modified by iterations of exposure to COVID-19 vaccines and to infections with increasingly divergent viral variants remains poorly understood, and is of particular relevance with the emergence of antigenically distinct VOCs. Omicron is the evolutionary most distant reported VOC to date, with a hitherto unprecedented number of amino acid alterations in its S glycoprotein, including at least 15 amino acid changes in the RBD and extensive changes in the N-terminal domain (NTD) (*15*). These alterations are predicted to affect most neutralizing antibody epitopes (*16*–*20*). In addition, Omicron is highly transmissible, has outcompeted Delta within weeks to become the dominant circulating VOC, and has given rise to multiple sublineages, starting with BA.1 and BA.2, that are spreading rapidly across the globe (*21*, *22*). New Omicron sublineages that harbor further alterations in the S glycoprotein continue to arise, with BA.4 and BA.5 deemed VOCs by the European Centre for Disease Prevention and Control (ECDC) on the 12^th^ May 2022 (*23*).

To characterize the effect of Omicron breakthrough infection on the magnitude and breadth of serum neutralizing activity and B_MEM_ cells, we studied blood samples from individuals that were double- or triple-vaccinated with BNT162b2, including cohorts that experienced breakthrough infection between November 2021 and mid-January 2022, a period when the BA.1 lineage was dominant in Germany (*24*). As an understanding of the antigen-specific B cell memory pool is a critical determinant of an individual’s ability to respond to newly emerging variants, our data will help to guide further vaccine development.

## RESULTS

### Cohorts and sampling

**Fig. 1.**
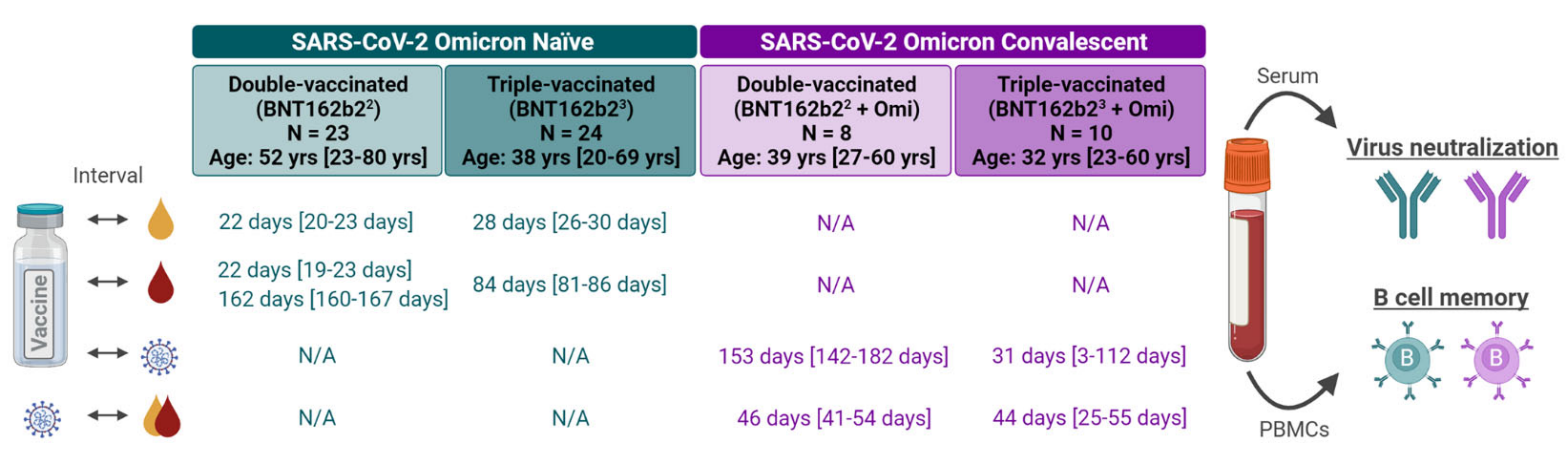
Cohorts, sampling and experimental setup. Blood samples were drawn from four cohorts: Omicron-naïve individuals double- or triple-vaccinated with BNT162b2 (light and dark green), and individuals double- or triple-vaccinated with BNT162b2 that subsequently had a breakthrough infection with Omicron (light and dark purple) at a time of BA.1 dominance. PBMCs (red) and sera (yellow) were isolated in the Omicron-naïve cohorts at the time-points indicated following their most recent vaccination; for convalescent cohorts, the time from their most recent vaccination to Omicron infection, and infection to PBMC and serum isolation are indicated in purple text. All values are specified as median [range]. The age/gender composition of cohorts is further detailed in Tables S4 and S10. Serum neutralizing capacity was assessed using pseudovirus and live virus neutralization tests. SARS-CoV-2 S glycoprotein-specific B_MEM_ cells were assessed via a flow cytometry-based B cell phenotyping assay using bulk PBMCs. N/A, not applicable. Schematic was created with BioRender.com.

Blood samples were sourced from the biosample collection of BNT162b2 vaccine trials, and a biobank of prospectively collected samples from vaccinated individuals with subsequent SARS-CoV-2 Omicron breakthrough infection experienced in a period of Omicron sublineage BA.1 dominance, and we therefore refer to “BA.1 breakthrough infection” herein. Samples were selected to investigate biomarkers in four independent groups, namely individuals who were (i) double- or (ii) triple-vaccinated with BNT162b2 without a prior or breakthrough infection at the time of sample collection (BNT162b2^2^, BNT162b2^3^) and individuals who were (iii) double- or (iv) triple-vaccinated with BNT162b2 and who experienced breakthrough infection with the SARS-CoV-2 Omicron variant after a median of approximately 5 months or 4 weeks, respectively (BNT162b2^2^ + Omi, BNT162b2^3^ + Omi). Median ages of the cohorts were similar (32-39 years), except for the BNT162b2^2^ cohort, which had a mildly increased median age of 52, albeit with only two individuals >65 yrs of age. Immune sera were used to characterize Omicron infection-associated changes to the magnitude and the breadth of serum neutralizing activity. PBMCs were used to characterize the VOC-specificity of peripheral B_MEM_ cells recognizing the respective full-length SARS-CoV-2 S glycoprotein or its RBD (Fig. 1, Tables S1-S3).

### Omicron BA.1 breakthrough infection after BNT162b2 vaccination induces broad neutralization against Omicron BA.1, BA.2 and other VOCs, but not against BA.4 and BA.5

To evaluate the neutralizing activity of immune sera, we used two orthogonal test systems: a well-characterized pseudovirus neutralization test (pVNT) (*25*, *26*) to investigate the breadth of inhibition of virus entry in a propagation-deficient set-up, as well as a live SARS-CoV-2 neutralization test (VNT) designed to evaluate neutralization during multicycle replication of authentic virus with the antibodies maintained throughout the entire test period. For the former, we applied pseudoviruses bearing the S glycoproteins of SARS-CoV-2 Wuhan, Alpha, Beta, Delta, Omicron BA.1, BA.2, and of the recently emerged Omicron sublineages BA.4 and BA.5 to assess neutralization breadth. As BA.4 and BA.5 share an identical S glycoprotein sequence, including key alterations L452R and F486V, we herein refer to them as BA.4/5. In addition, we assayed SARS-CoV (herein referred to as SARS-CoV-1) to detect potential pan-sarbecovirus neutralizing activity (*27*).

**Fig. 2.**
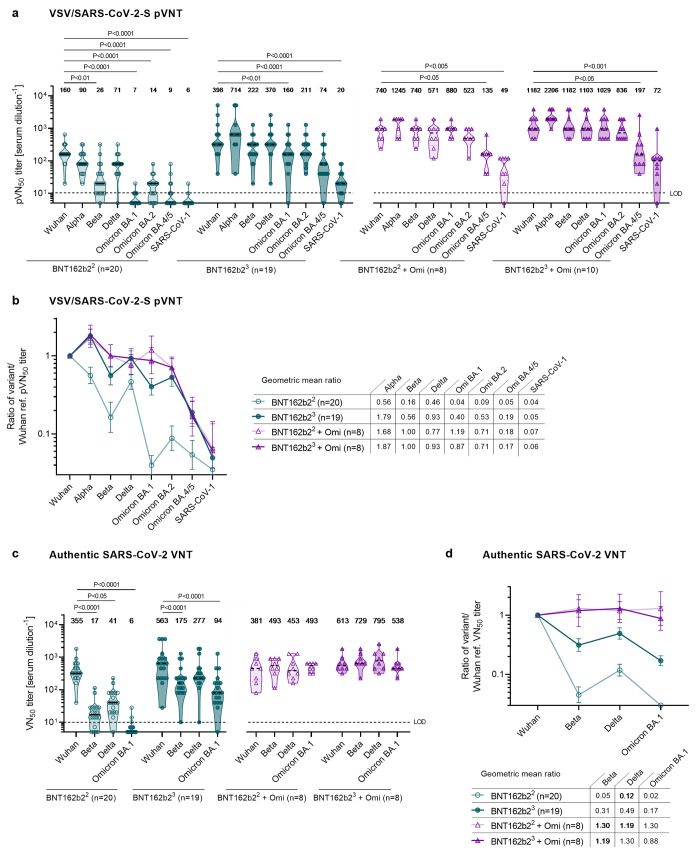
Omicron BA.1 breakthrough infection of BNT162b2 double- and triple-vaccinated individuals induces broad neutralization of Omicron BA.1, BA.2 and other VOCs, but not BA.4 and BA.5. Serum was drawn from double-vaccinated individuals (BNT162b2^2^) at 22 days after the second dose (green, open circles), from triple-vaccinated individuals (BNT162b2^3^) at 28 days after the third dose (green, closed circles), from double-vaccinated individuals with Omicron breakthrough infection (BNT162b2^2^ + Omi) at 46 days post-infection (purple, open triangles), and from triple-vaccinated individuals and Omicron breakthrough infection (BNT162b2^3^ + Omi) at 44 days post-infection (purple, closed triangles). Serum was tested in duplicate; 50% pseudovirus neutralization (pVN_50_) geometric mean titers (GMTs) (in a), the geometric mean ratio of SARS-CoV-2 variants of concern (VOCs) and SARS-CoV-1 pVN_50_ GMTs normalized against Wuhan pVN_50_ GMTs (in b), 50% virus neutralization (VN_50_) GMTs (in c), and the geometric mean ratio of SARS-CoV-2 variants of concern (VOCs) VN_50_ GMTs normalized against Wuhan VN_50_ GMTs (in d) were plotted. For titer values below the limit of detection (LOD), LOD/2 values were plotted. Values above violin plots represent the group GMTs. The nonparametric Friedman test with Dunn’s multiple comparisons correction was used to compare Wuhan neutralizing group GMTs with titers against the indicated variants and SARS-CoV-1. Multiplicity-adjusted p values are shown. (**a**) pVN_50_ GMTs against Wuhan, VOC and SARS-CoV-1 pseudovirus. (**b**) Group geometric mean ratios with 95% confidence intervals for all cohorts shown in a. (**c**) VN_50_ GMTs against live SARS-CoV-2 Wuhan, Beta, Delta and Omicron BA.1. (**d**) Group geometric mean ratios with 95% confidence intervals for all cohorts shown in c.

As reported previously (*25*, *28*, *29*), in Omicron-naïve double-vaccinated individuals 50% pseudovirus neutralization (pVN_50_) geometric mean titers (GMTs) of Beta and Delta VOCs were reduced, and neutralization of Omicron BA.1, BA.2 and BA.4/5 was virtually undetectable (Fig. 2a, fig. S1a, Table S4). In Omicron-naïve triple-vaccinated individuals, pVN_50_ GMTs against all tested VOCs were substantially higher with robust neutralization of Alpha, Beta and Delta. While GMTs against Omicron BA.1 and BA.2 were already considerably lower as compared with Wuhan (GMT 160 and 211 vs 398), neutralizing activity against Omicron BA.4/5 was even further reduced (GMT 74), corresponding to a 5-fold lower titer as compared to the Wuhan strain (Fig. 2a, fig. S1a, Table S5).

Omicron BA.1 breakthrough infection had a marked effect on magnitude and breadth of the neutralizing antibody response of both double- and triple-vaccinated individuals, with slightly higher pVN_50_ GMTs observed in the triple-vaccinated individuals (Fig. 2a, fig. S1b, Table S6). The pVN_50_ GMT of double-vaccinated individuals with breakthrough infection against Omicron BA.1, BA.2 and BA.4/5 was more than 100-fold, 35-fold and 15-fold above the GMTs of Omicron-naïve double-vaccinated individuals. Immune sera from double-vaccinated individuals with BA.1 breakthrough infection had broad neutralizing activity against Omicron BA.1, BA.2 and previous SARS-CoV-2 VOCs, with higher pVN_50_ GMTs against Beta and Delta than observed in Omicron-naïve triple-vaccinated individuals (GMT 740 vs. 222 and 571 vs. 370). In contrast, Omicron BA.1 breakthrough infection had only a minor boost effect on neutralization of BA.4/5 with pVN_50_ GMTs against Omicron BA.4/5 being significantly below those against Wuhan (GMT 135 vs. 740).

We observed a similar pattern when studying the neutralization of these variants with BA.1 convalescent and control sera from triple-vaccinated individuals. BA.1 convalescent sera exhibited high pVN_50_ GMTs against the previous SARS-CoV-2 VOCs, including Beta (1182), Omicron BA.1 (1029), and BA.2 (836) that were close to the Wuhan reference (1182). Omicron BA.1 breakthrough infection only moderately increased neutralization of BA.4/5 in triple-vaccinated individuals with pVN_50_ GMTs of 197, remaining 6-fold lower than against the Wuhan strain.

Of note, in all cohorts, neutralizing titers against BA.4/5 were closer to the low level observed against the phylogenetically more distant SARS-CoV-1 than that seen against Wuhan (Fig. 2a, Table S4 to S6). Looking at the ratios of SARS-CoV-2 VOC and SARS-CoV-1 pVN_50_ GMTs normalized against Wuhan, it is remarkable that breakthrough infection with Omicron BA.1 does not lead to more efficient cross-neutralization of Omicron BA.4/5 in double- and triple-vaccinated individuals as compared with triple-vaccinated Omicron-naïve individuals (Fig. 2b).

Authentic live SARS-CoV-2 virus neutralization assays conducted with Wuhan, Beta, Delta and Omicron BA.1 confirmed the observation that BA.1 breakthrough infection boosted broad immunity against BA.1 and previous SARS-CoV-2 VOCs (Fig. 2c, fig. S1c, d, Tables S7 to S9). In BNT162b2 double- and triple-vaccinated individuals, Omicron BA.1 breakthrough infection was associated with a strongly increased neutralizing activity against Omicron BA.1, with 50% virus neutralization (VN_50_) GMTs in the same range as against the Wuhan strain (Fig. 2c; GMT 493 vs. 381 and GMT 538 vs. 613). Similarly, BA.1 convalescent double- and triple-vaccinated individuals showed comparable levels of neutralization against other variants as well (e.g., GMT 493 and 729 against Beta), indicating a wide breadth of neutralizing activity, a finding further supported by the calculated ratios of SARS-CoV-2 VOC VN_50_ GMTs normalized against the Wuhan strain (Fig. 2d). While double- and to a lesser extent also triple-BNT162b2 vaccinated Omicron-naïve individuals displayed reduced neutralization proficiency against VOCs, neutralization activity of Omicron BA.1 convalescent subjects reached almost the same range of high performance against all live SARS-CoV-2 variant strains tested. Likewise, Omicron BA.1 breakthrough infection similarly augmented broad neutralization in individuals vaccinated with other approved COVID-19 vaccines or heterologous regimens, but with significantly reduced potency against Omicron BA.4/5 (fig. S2, Table S11). In aggregate, these data demonstrate that Omicron BA.1 breakthrough infection of vaccine-experienced individuals mediates broadly neutralizing activity against BA.1, BA.2 and several previous SARS-CoV-2 variants, but not for BA.4/5.

### B_MEM_ cells of BNT162b2 double- and triple-vaccinated individuals broadly recognize VOCs and are further boosted by Omicron BA.1 breakthrough infection

**Fig. 3.**
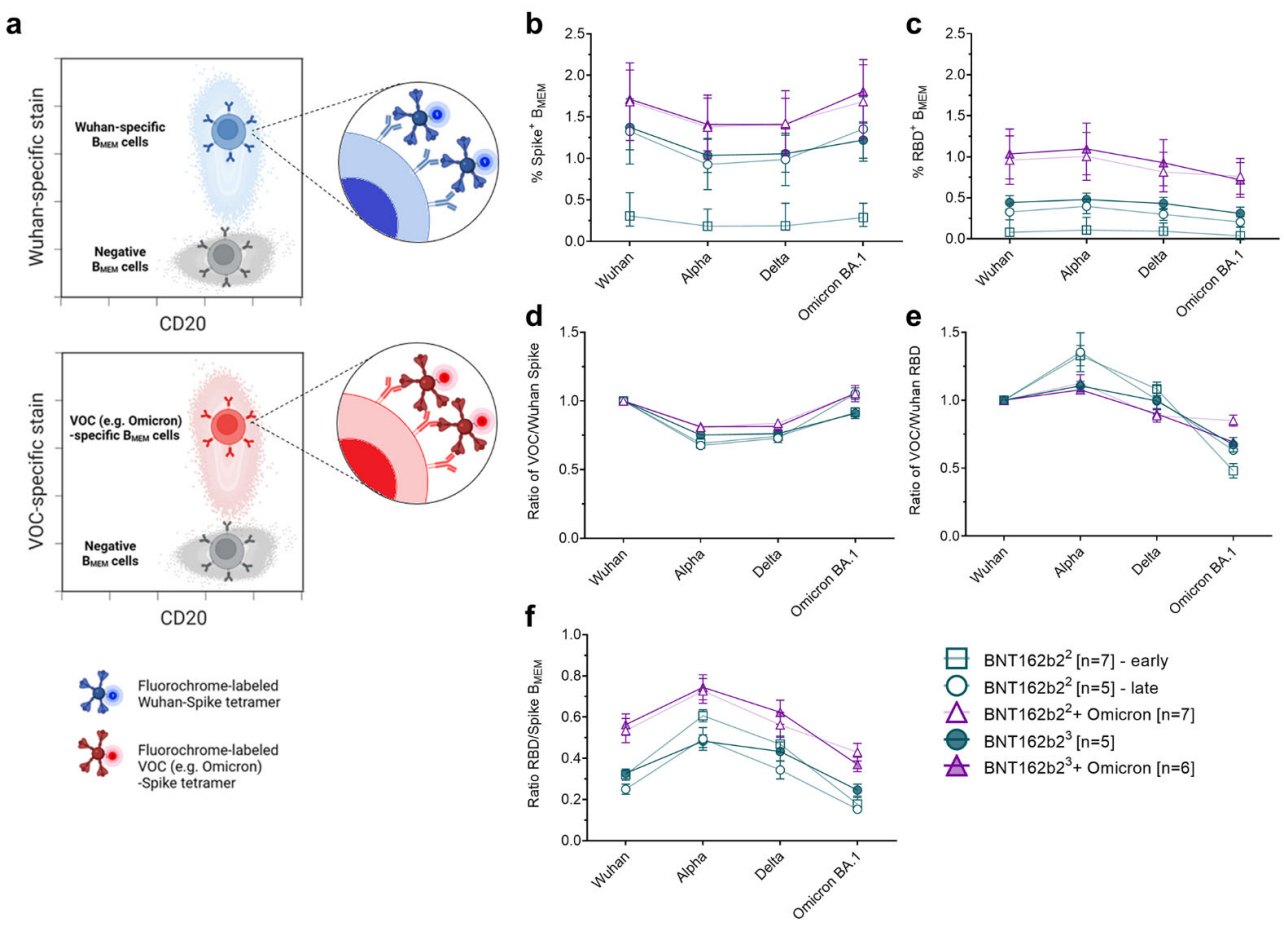
B_MEM_ cells of individuals double- and triple-vaccinated with BNT162b2 broadly recognize VOCs and are further boosted by Omicron BA.1 breakthrough infection. PBMC samples from double-vaccinated individuals (BNT162b2^2^) at 22 days after the second dose (green, open squares) and 5 months after the second dose (green, open circles), from triple-vaccinated individuals (BNT162b2^3^) at 84 days after the third dose (green, closed circles), from double-vaccinated individuals with Omicron breakthrough infection (BNT162b2^2^ + Omi) at 46 days post-infection (purple, open triangles), and from triple-vaccinated individuals with Omicron breakthrough infection (BNT162b2^3^ + Omi) at 44 days post-infection (purple, closed triangles) were analyzed via flow cytometry for SARS-CoV-2-specific B_MEM_ cell (B_MEM_ – CD3^-^CD19^+^CD20^+^IgD^-^CD38^int/low^) frequencies via B cell bait staining. (**a**) Schematic of one-dimensional staining of B_MEM_ cells with fluorochrome-labeled SARS-CoV-2 S glycoprotein tetramer bait allowing discrimination of variant recognition. Frequencies of Wuhan or VOC full-length S glycoprotein- (**b**) and RBD- (**c**) specific B_MEM_ cells for the four groups of individuals were analyzed. Variant-specific B_MEM_ cell frequencies were normalized to Wuhan frequencies for S glycoprotein (**d**) and RBD (**e**) binding. (**f**) The frequency ratios of RBD protein-specific B_MEM_ cells over full-length S glycoprotein-specific B_MEM_ cells are depicted. Mean values and standard error of the mean (SEM) are shown in (b)-(f). Statistical comparisons between individuals of each group presented in (b) and (c) were performed using the nonparametric Friedman test with Dunn’s multiple comparisons correction presented in fig S4 (a-j). n = number of individuals per group. Schematic in (a) was created with BioRender.com.

Next, we investigated the phenotype and quantity of SARS-CoV-2 S glycoprotein-specific B cells in these individuals. To this aim, we employed flow cytometry-based B cell phenotyping assays for differential detection of variant-specific S glycoprotein-binding B cells in bulk PBMCs. We found that all S glycoprotein- and RBD-specific B cells in the peripheral blood were of a B_MEM_ phenotype (B_MEM_; CD20^high^CD38^int/neg^, fig. S3a). Antigen-specific plasmablasts or naïve B cells were not detected. The assays allowed us to identify B_MEM_ cells recognizing the S glycoprotein (fig S3b) or RBD (fig S3c) of SARS-CoV-2 Wuhan, Alpha, Delta and Omicron BA.1 variants (Fig. 3a, data supporting bait specificity presented in fig S3d-f).

As expected, the overall frequency of antigen-specific B_MEM_ cells varied across the different groups. Consistent with prior reports (*30*), the frequency of B_MEM_ cells in Omicron-naïve double-vaccinated individuals was low at an early time point after vaccination and increased over time: At 5 months as compared to 3 weeks after the second BNT162b2 dose, S glycoprotein-specific B_MEM_ cells almost quadrupled, and RBD-specific ones tripled across all VOCs thereby reaching quantities similar to those observed in Omicron-naïve triple-vaccinated individuals (Fig. 3b, c, fig. S4a-c, Table S12).

Double or triple BNT162b2-vaccinated individuals with a SARS-CoV-2 Omicron BA.1 breakthrough infection exhibited a strongly increased frequency of S glycoprotein-specific B_MEM_ cells, which was higher than those of Omicron-naïve triple-vaccinated individuals (Fig. 3b, d fig. S4d, e, k, l).

In all groups, including Omicron-naïve and Omicron BA.1 infected individuals, B_MEM_ cells against Omicron BA.1 S glycoprotein were detectable at frequencies comparable to those against Wuhan and other tested VOCs (Fig. 3b, d), whereas the frequency of B_MEM_ cells against Omicron BA.1 RBD was slightly lower compared to the other variants (Fig. 3c, e, fig. S4f-j, m, n). We then compared the ratios of RBD- to S glycoprotein-binding B_MEM_ cells within the different groups and found that they are biased toward S glycoprotein recognition for the Omicron BA.1 VOC, particularly in the Omicron-naïve groups (Fig. 3f). In the Omicron BA.1 convalescent groups this ratio was higher, indicating that an Omicron BA.1 breakthrough infection improved Omicron BA.1 RBD recognition.

### Omicron BA.1 breakthrough infection after BNT162b2 vaccination boosts B_MEM_ cells against epitopes broadly conserved across S glycoproteins of Wuhan and other VOCs.

**Fig. 4.**
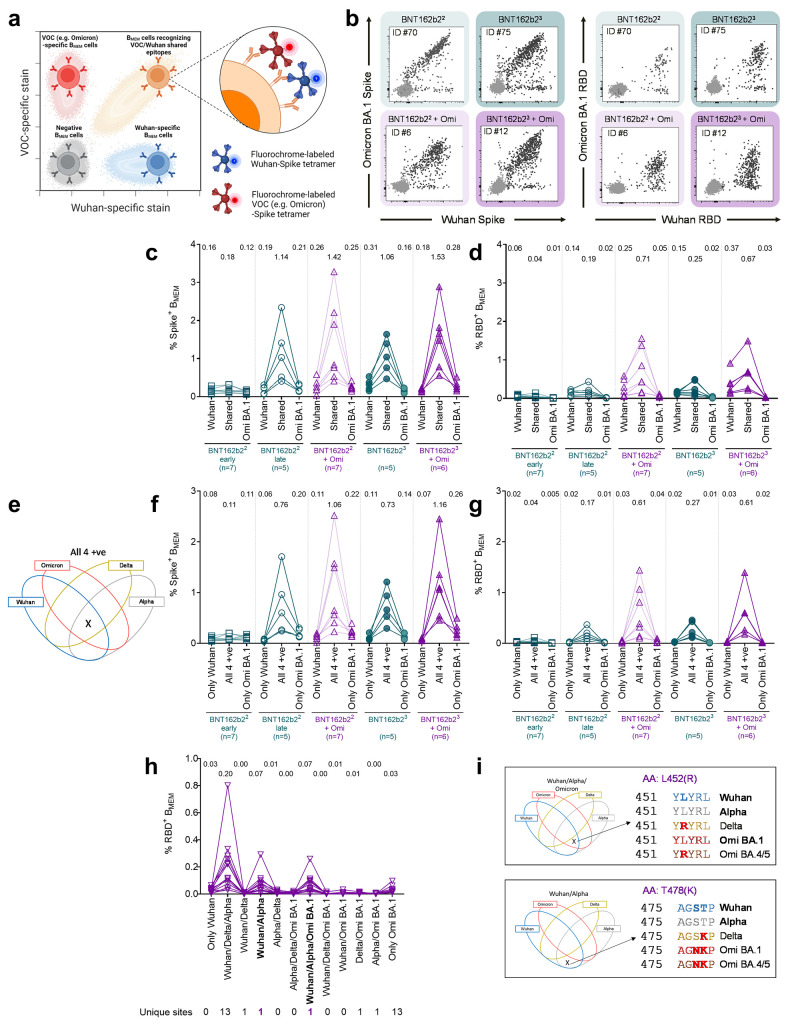
Omicron BA.1 breakthrough infection of BNT162b2 double- and triple-vaccinated individuals primarily boosts B_MEM_ against conserved epitopes shared broadly between S glycoproteins of Wuhan and other VOCs rather than strictly Omicron S-specific epitopes. PBMC samples from double-vaccinated individuals (BNT162b2^2^) at 22 days after the second dose (green, open squares) and 5 months after the second dose (green, open circles), from triple-vaccinated individuals (BNT162b2^3^) at 84 days after the third dose (green, closed circles), from double-vaccinated individuals with Omicron breakthrough infection (BNT162b2^2^ + Omi) at 46 days post-infection (purple, open triangles), and from triple-vaccinated individuals with Omicron breakthrough infection (BNT162b2^3^ + Omi) at 44 days post-infection (purple, closed triangle) were analyzed via flow cytometry for SARS-CoV-2-specific memory B cells (B_MEM_ – CD3^-^CD19^+^CD20^+^IgD^-^CD38^int/low^) frequencies via B cell bait staining (**a**). (**b**) Representative flow plots of Omicron and Wuhan S glycoprotein- and RBD-binding for each of the four groups of individuals investigated. Frequencies of B_MEM_ binding Omicron, Wuhan, or both (shared) full-length S glycoprotein (**c**) or RBD (**d**) for Omicron-experienced and naïve BNT162b2 double and triple vaccinees are shown. (**e**) Venn diagrams visualizing the combinatorial (Boolean) gating strategy to identify cross-reactive B_MEM_ recognizing all four variants simultaneously (All 4 +ve) and B_MEM_ recognizing only Omicron (only Omi) or only Wuhan (only Wuhan) S glycoproteins. Frequencies for these three B_MEM_ sub-groups were compared for full-length S glycoprotein (**f**) and RBD (**g**) in the four different groups of individuals investigated. RBD variant recognition pattern by B_MEM_ was assessed through Boolean flow cytometric gating strategy and frequencies recognizing combinations of Wuhan and/or variant RBDs are displayed in (**h**), for all Omicron convalescent subjects (double and triple vaccinees pooled, n=13). (**i**) Conserved site within the RBD domain recognized by RBD-specific B_MEM_ after Omicron break-through infection. Mean values are indicated in c, d, f, g, and h. n = number of individuals per group. Results for the nonparametric Friedman test with Dunn’s multiple comparisons correction testing for significance within treatment groups against *shared* (c,d) and *all 4+ve* (f,g) are presented in Table S13. Schematic in (a) was created with BioRender.com.

Our findings imply that Omicron BA.1 infection in vaccinated individuals boosts not only neutralizing activity and B_MEM_ cells against Omicron BA.1, but broadly augments immunity against various VOCs. To investigate the specificity of antibody responses at a cellular level, we performed multi-parameter analyses of B_MEM_ cells stained with fluorescently labeled variant-specific S or RBD proteins. By applying a combinatorial gating strategy, we sought to distinguish between B_MEM_ cell subsets that could identify epitopes specific to a single variant only (either Wuhan, Alpha, Delta or Omicron BA.1) versus those that could identify epitopes shared by any given combination of these variants (Fig. 4a, fig. S3).

In a first analysis, we evaluated B_MEM_ cell recognition of Wuhan and Omicron BA.1 S and RBD proteins (Fig. 4b-d). Staining with full length S glycoproteins showed that the largest proportion of B_MEM_ cells from Omicron-naïve double-vaccinated individuals, and even more predominantly from triple-vaccinated individuals were directed against epitopes shared by both Wuhan and Omicron BA.1 SARS-CoV-2 variants. Consistent with the fact that vaccination with BNT162b2 can elicit immune responses against Wuhan epitopes that do not recognize the corresponding altered epitopes in the Omicron BA.1 S glycoprotein (Fig. 4b, c, fig. S5a), we found in most individuals a smaller but clearly detectable proportion of B_MEM_ cells that recognized only Wuhan S glycoprotein or RBD. Consistent with the lack of exposure, almost no B_MEM_ cells binding exclusively to Omicron BA.1 S or RBD protein were detected in these Omicron-naïve individuals.

In Omicron BA.1 convalescent individuals, frequencies of B_MEM_ cells recognizing S glycoprotein epitopes shared between Wuhan and Omicron BA.1 were considerably higher than in the Omicron-naïve ones (Fig. 4b, c). This was particularly pronounced for double-vaccinated individuals. In most of these subjects, we also found a small proportion of exclusively Wuhan S glycoprotein-specific B_MEM_ cells, as well as a moderately lower frequency of exclusively Omicron BA.1 variant S glycoprotein-specific B_MEM_ cells (fig. S5a).

A slightly different pattern was observed by B cell staining with labeled RBD proteins (Fig. 4b, d, Fig. S5b). Again, Omicron BA.1 breakthrough infection of double-/triple-vaccinated individuals was found to primarily boost B_MEM_ cells reactive against conserved epitopes. A moderate boost of Wuhan-specific reactivities was observed; however, we detected only small populations of B_MEM_ cells specific to the Omicron BA.1-RBD in the tested individuals (Fig. 4d, fig. S5b).

Next, we employed the combinatorial gating approach to identify the subsets of S glycoprotein or RBD binding B_MEM_ cells that either bind exclusively to Wuhan or Omicron BA.1, or to common epitopes conserved broadly throughout all four variants, Wuhan, Alpha, Delta and Omicron BA.1 (Fig. 4e). Across all four cohorts, we found that the frequency of B_MEM_ cells recognizing S glycoprotein-conserved epitopes accounted for the largest fraction of the pool of S glycoprotein-binding B_MEM_ cells (Fig. 4f, all 4+ve). The S glycoprotein of the Wuhan strain does not have an exclusive amino acid change that distinguishes it from the S glycoproteins of the Alpha, Delta, or Omicron BA.1 VOCs. Accordingly, we hardly detected B_MEM_ cells exclusively recognizing the Wuhan S glycoprotein in any individual (Fig. 4f). In several individuals with Omicron BA.1 breakthrough infection, we detected a small proportion of B_MEM_ cells that bound exclusively to Omicron BA.1 S glycoprotein (Fig. 4f), whereas almost none of the individuals displayed a strictly Omicron BA.1 RBD-specific response (Fig. 4 g). Our findings indicate that Omicron BA.1 breakthrough infection in vaccinated individuals primarily expands a broad B_MEM_ cell repertoire against conserved S glycoprotein and RBD epitopes rather than inducing large numbers of Omicron BA.1-specific B_MEM_ cells.

To further dissect the nuances of this response, we characterized the B_MEM_ subsets directed against the RBD in both double- and triple-vaccinated Omicron BA.1 convalescent individuals. We used the combinatorial Boolean gating approach to discern B_MEM_ cells with distinct binding patterns in the spectrum of strictly variant-specific and common epitopes shared by several variants. Multiple sequence alignment revealed that the Omicron BA.1 RBD diverges from the RBD sequence regions conserved in Wuhan, Alpha, and Delta by 13 single amino acid alterations (fig. S6). The most prominent B_MEM_ cell population that we detected in BA.1 convalescent individuals recognized Wuhan, Alpha as well as the Delta RBDs, but not Omicron BA.1 RBD (Fig. 4h). Contrary to expectations, the population of B_MEM_ cells exclusively reactive with Omicron BA.1 RBD was small in most of those individuals. We did not detect B_MEM_ cells that exclusively recognized epitopes shared by both the Omicron BA.1 and Alpha RBDs, or by the Omicron BA.1 and Delta RBDs.

Furthermore, in all individuals we identified two additional subsets of RBD-specific B_MEM_ cells, (in bold in Fig. 4h). One subset was characterized by binding to the RBDs of Wuhan, Alpha as well as Omicron BA.1, but not the Delta RBD. The other population exhibited binding to Wuhan and Alpha but not Omicron BA.1 or Delta RBD. Sequence alignment identified L452R as the only RBD alteration unique for Delta that is not shared by the other 3 variant RBDs (Fig. 4i top). Similarly, the only RBD site conserved in Wuhan and Alpha but altered in Delta and Omicron BA.1 was found to be T478K (Fig. 4i bottom). Both L452R and T478K alterations are known to be associated with the evasion of vaccine induced neutralizing antibody responses (*31*, *32*). Position L452 is in fact mutated in the recently emerged SARS-CoV-2 Omicron sublineages BA.4 and BA.5 (*33*). Of note, only minor B_MEM_ cell frequencies were detected in those combinatorial subgroups in which multiple sequence alignment failed to identify unique epitopes in the RBD sequence (e.g., Wuhan only or Wuhan and Omicron BA.1, but not Alpha, Delta). These observations indicate that the B_MEM_ cell response against RBD is driven by specificities induced through prior vaccination with BNT162b2 and not substantially redirected against new RBD epitopes displayed by the infecting Omicron BA.1 variant.

## DISCUSSION

SARS-CoV-2 Omicron BA.1 is a partial immune escape variant with an unprecedented number of amino acid alterations in the S glycoprotein at sites of neutralizing antibody binding (*15*). Neutralizing antibody mapping and molecular modeling studies strongly support the functional relevance of these alterations (*20*, *34*), that is confirmed by the fact that double-vaccinated individuals have no detectable neutralizing activity against SARS-CoV-2 Omicron BA.1 (*25*, *35*).

In line with concurrently published reports (*36*, *37*), we show that Omicron BA.1 breakthrough infection of BNT162b2 vaccinated individuals augments broadly neutralizing activity against Omicron BA.1, BA.2 and previous VOCs to similar levels observed against the Wuhan strain. However, neutralization of the latest Omicron sublineages BA.4 and BA.5 was not enhanced, with titers rather comparable to those against the phylogenetically more distant SARS-CoV-1. While our study focused on individuals vaccinated with the BNT162b2 mRNA vaccine, in individuals vaccinated with CoronaVac similar observations suggest that Omicron BA.4/5 can bypass BA.1 infection-mediated boosting of humoral immunity (*33*).

Our study provides insights into how immunity against multiple variants is achieved and why Omicron BA.4 and BA.5 sublineages can partially escape neutralization. It suggests that initial exposure to the Wuhan strain S glycoprotein may have shaped the formation of B_MEM_ cells and imprinted against novel B_MEM_ cell responses recognizing epitopes distinctive for the Omicron BA.1 variant. Omicron BA.1 breakthrough infection in BNT162b2-vaccinated individuals primarily expands a broad B_MEM_ cell repertoire against conserved S glycoprotein and RBD epitopes, rather than inducing strictly Omicron BA.1-specific B_MEM_ cells. Similar observations have been reported from vaccinated individuals who experienced breakthrough infections with the Delta variant and with the Omicron BA.1 sublineage (*3*, *33*).

As compared to the immune response induced by a homologous vaccine booster, an Omicron BA.1 breakthrough infection leads to a more substantial increase in antibody neutralization titers against Omicron and a robust cross-neutralization of many SARS CoV-2 variants. These effects are particularly striking in double-vaccinated individuals.

Three findings may point to potentially complementary and synergistic underlying mechanisms. The first is induction of broadly neutralizing antibodies. We found that the majority of sera from Omicron BA.1-convalescent but not from Omicron-naïve vaccinated individuals robustly neutralize previous SARS-CoV-2 VOCs including BA.1 and BA.2, and to a far lesser extent also SARS-CoV-2 Omicron BA.4/5 and SARS-CoV-1. This indicates that Omicron BA.1 infection in vaccinated individuals stimulates B_MEM_ cells that produce neutralizing antibodies against S glycoprotein epitopes conserved in the SARS-CoV-2 variants up to and including Omicron BA.2, but that have mostly been lost in BA.4/5 and are for the most part not shared by SARS-CoV-1. Over the last two years, broadly cross-neutralizing antibodies have been isolated from both SARS-CoV-2 and SARS-CoV-1 convalescent and/or vaccinated individuals (*20*, *27*, *38*) and are known to target highly conserved S glycoprotein domains (*39*, *40*). The greater antigenic distance of the Omicron BA.1 S glycoprotein from earlier SARS-CoV-2 strains may promote targeting of conserved subdominant neutralizing epitopes as recently described to be located, e.g., in cryptic sites within the RBD distinct from the receptor-binding motif (*41*, *42*) or in the membrane proximal S glycoprotein subunit designated S2 (*43*–*45*).

The second finding is a bias toward RBD-specific B_MEM_ cell responses. Omicron BA.1-infected individuals appear to have a significantly higher RBD/S glycoprotein-specific B_MEM_ cell ratio as compared to vaccinated Omicron-naïve individuals. Omicron BA.1 carries multiple S glycoprotein alterations such as del69/70 and del143-145 in key neutralizing antibody binding sites of the NTD that dramatically reduce the targeting surface for memory B cell responses in this region. Although the Omicron BA.1 RBD harbors multiple alterations, there are some unaffected neutralizing antibody binding sites left (*20*). An expansion of B_MEM_ cells that produce neutralizing antibodies against RBD epitopes that are not altered in Omicron BA.1, such as those at position L452 as indicated in our study, could help to rapidly restore neutralization of the BA.1 and BA.2 variants. Importantly, the strong neutralization of Omicron BA.1 and BA.2 should not mask the fact that the neutralizing B_MEM_ immune response in Omicron BA.1 convalescent vaccinated individuals is driven by a smaller number of epitopes. The significantly reduced neutralizing activity against the Omicron BA.4/5 pseudovirus, which harbors the additional alterations L452R and F486V in the RBD, demonstrates the mechanism of immune evasion by loss of the few remaining conserved epitopes. Meanwhile, further sublineages with L452 alterations (e.g., BA.2.12.1) are being reported to evade humoral immunity elicited by BA.1 breakthrough infection (*33*).

The third finding is an overall increase of S glycoprotein-specific B_MEM_ cells. Omicron BA.1-convalescent double-vaccinated individuals appear to have a higher frequency of B_MEM_ cells and higher neutralizing antibody titers against previous VOCs as compared to triple-vaccinated individuals. Studies on other VOCs have not shown breakthrough infections in double-vaccinated individuals to be superior to a third vaccine dose in eliciting neutralizing activity (*4*, *36*). This may be explained by poor neutralization of the partial escape Omicron BA.1 variant in the initial phase of infection, which may result in greater or prolonged antigen exposure of the immune system to the altered S glycoprotein.

In aggregate, our results suggest that despite potential imprinting of the immune response by previous vaccination, the preformed B cell memory pool can be refocused and quantitatively remodeled by exposure to heterologous S glycoproteins to allow neutralization of variants that evade a previously established neutralizing antibody response. However, our data also suggest that the immunity in the early stage of Omicron BA.1 infection in vaccinated individuals is based on recognition of conserved epitopes and is narrowly focused on a small number of neutralizing sites that are not altered in Omicron BA.1 and BA.2. Such a narrow immune response bears a high risk that those few epitopes may be lost by acquisition of further alterations in the course of the ongoing evolution of Omicron and may result in immune escape, as being experienced with sublineages BA.2.12.1, BA.4 and BA.5 (*33*, *46*). Importantly, Omicron BA.1 breakthrough infection does not appear to reduce the overall spectrum of (Wuhan) S glycoprotein-specific memory B cells, as memory B cells that do not recognize Omicron BA.1 S remain detectable in blood at similar frequencies. We consistently detected Wuhan-specific (non-Omicron BA.1 reactive) B_MEM_ cells in Omicron BA.1 breakthrough infected individuals at levels similar to those in Omicron-naïve double-/triple-vaccinated individuals. Our data therefore suggest an increase of the total B_MEM_ cell repertoire by selective amplification of B_MEM_ cells that recognize shared epitopes.

Our findings raise a number of questions, e.g., to what extent induced B_MEM_ responses are functional and directed against neutralizing domains. A recent study examined more than 600 neutralizing antibodies isolated from triple-CoronaVac vaccinated individuals who subsequently experienced BA.1 breakthrough infection. Consistent with our findings, the study showed that BA.1 infection in vaccinated individuals primarily retrieves Wuhan S glycoprotein-induced B cell memory and elicits cross-reactive neutralizing antibodies against RBD epitopes that neutralize both the ancestral SARS-CoV-2 Wuhan as well as the Omicron BA.1 variant (*33*). Also, it is not yet clear whether the B_MEM_ cells against conserved epitopes that we observed after Omicron BA.1 breakthrough infection are newly recruited cross-reactive naïve B cells, or rather expanded from the pre-existent memory B cell pool. A recent study investigating a third vaccine booster suggests that both mechanisms are relevant (*47*). Further, we cannot exclude that strictly Omicron-BA.1 specific B_MEM_ cells are in fact being efficiently generated but had just not been exported from the germinal center at the time point of our analysis. These questions can be addressed by comprehensive studies of the B cell repertoire at later time points (> 3months) after breakthrough infection, including BCR repertoire analysis by single cell Ig gene sequencing of antigen-specific B_MEM_ cells, extended to the cloning, expression and characterization of monoclonal antibodies with regard to specificity, functional properties, and affinity.

Our findings are based on retrospective analyses of samples derived from different studies. Therefore, the sample sizes were relatively small and cohorts were not fully adjusted with regard to immunization intervals, sampling time points and demographic characteristics such as age and sex of individuals. Another limitation is that the analysis was restricted to B_MEM_ cells; long-lived bone marrow-derived plasma cells (BMPCs), which are known to be BNT162b2 vaccination induced (*48*), were not investigated as they cannot be cryopreserved.

A key motivation for our study was to inform our vaccine adaptation program. We expect that the currently ongoing vaccine adaptations to the Omicron BA.1 S glycoprotein, similar to the Omicron BA.1 breakthrough infection that we studied, may reshape the B cell memory repertoire and provide broad protection against previous VOCs. However, given the rapid evolution of SARS-CoV-2, other sublineages of Omicron that antigenically deviate from BA.1 even more than the immune escape variants BA.4/5, may have emerged by the time of potential authorization of those vaccines later this year. In a pandemic in which a highly transmissible VOC feeds dynamic and rapid evolution of altered variants, an effective strategy may be to leverage the full potential of mRNA vaccine technology, which allows production and release of new vaccines in less than three months. To enable adapted vaccines that truly reflect relevant VOCs at licensure, it would be prudent to build on decades of experience with seasonal influenza vaccines and implement timely, rapid licensure procedures that use the latest epidemiologic data to select COVID-19 vaccine strains.

## MATERIALS AND METHODS

### Study design

The objective of this study was to investigate the effect of Omicron BA.1 breakthrough infection on the cross-variant neutralization capacity of human sera, and how repeat SARS-CoV-2 antigen exposure modulates B_MEM_ cell specificity in individuals vaccinated with BNT162b2. We compared immune responses in Omicron-naïve individuals double- or triple-vaccinated with BNT162b2, to that of individuals double- or triple-vaccinated with BNT162b2 with a confirmed subsequent breakthrough infection with Omicron during a period of Omicron sublineage BA.1 dominance. Serum neutralizing capability was characterized using live and pseudovirus neutralization assays, and flow cytometry was used to detect and characterize SARS-CoV-2-specific B cells in bulk PBMCs. Cross neutralization of variants was further characterized in a cohort vaccinated with other approved COVID-19 vaccines or mixed regimens, that experienced subsequent Omicron breakthrough infection. All participants had no documented history of SARS-CoV-2 infection prior to vaccination.

### Recruitment of participants and sample collection

Individuals from the SARS-CoV-2 Omicron-naïve BNT162b2 double-vaccinated (BNT162b2^2^) and triple-vaccinated (BNT162b2^3^) cohorts provided informed consent as part of their participation in a clinical trial (the Phase 1/2 trial BNT162-01 [NCT04380701] (*29*), the Phase 2 rollover trial BNT162-14 [NCT04949490], or as part of the BNT162-17 [NCT05004181] trial).

Participants from the SARS-CoV-2 Omicron convalescent double- and triple-vaccinated cohorts (BNT162b2^2^ + Omi and BNT162b2^3^ + Omi cohorts, respectively) and individuals vaccinated with other approved COVID-19 vaccines or mixed regimens with subsequent Omicron breakthrough infection were recruited from University Hospital, Goethe University Frankfurt as part of a research program that recruited patients who had experienced Omicron breakthrough infection following vaccination for COVID-19, to provide blood samples and clinical data for research. Omicron infections were confirmed with variant-specific PCR between November 2021 and mid-January 2022, at a time when sublineage BA.1 was dominant (*24*). The infections of 7 participants in this study were further characterized by genome sequencing, 5 of whom were in the BNT162b2-vaccinated cohorts, and 2 in the cohort with participants vaccinated with other approved COVID-19 vaccines or mixed regimens. In all 7 cases, genome sequencing confirmed Omicron BA.1 infection (Table S3 and S10).

Participants were free of symptoms at the time of blood collection. The study protocol for this research program was approved by the Ethics Board of the University Hospital, Goethe University Frankfurt (No. 2021-560). Demographic and clinical information for all participants as well as sampling timepoints are provided in Tables S1-S3 and S10, and Fig. 1. Serum was isolated by centrifugation 2000 × g for 10 min and cryopreserved until use. Li-Heparin blood samples were isolated by density gradient centrifugation using Ficoll-Paque PLUS (Cytiva) and were subsequently cryopreserved until use.

### VSV-SARS-CoV-2 S variant pseudovirus generation

A recombinant replication-deficient vesicular stomatitis virus (VSV) vector that encodes green fluorescent protein (GFP) and luciferase instead of the VSV-glycoprotein (VSV-G) was pseudotyped with SARS-CoV-1 S glycoprotein (UniProt Ref: P59594) and with SARS-CoV-2 S glycoprotein derived from either the Wuhan reference strain (NCBI Ref: 43740568), the Alpha variant (alterations: Δ69/70, Δ144, N501Y, A570D, D614G, P681H, T716I, S982A, D1118H), the Beta variant (alterations: L18F, D80A, D215G, Δ242–244, R246I, K417N, E484K, N501Y, D614G, A701V), the Delta variant (alterations: T19R, G142D, E156G, Δ157/158, K417N, L452R, T478K, D614G, P681R, D950N), the Omicron BA.1 variant (alterations: A67V, Δ69/70, T95I, G142D, Δ143-145, Δ211, L212I, ins214EPE, G339D, S371L, S373P, S375F, K417N, N440K, G446S, S477N, T478K, E484A, Q493R, G496S, Q498R, N501Y, Y505H, T547K, D614G, H655Y, N679K, P681H, N764K, D796Y, N856K, Q954H, N969K, L981F), the Omicron BA.2 variant (alterations: T19I, Δ24-26, A27S, G142D, V213G, G339D, S371F, S373P, S375F, T376A, D405N, R408S, K417N, N440K, S477N, T478K, E484A, Q493R, Q498R, N501Y, Y505H, D614G, H655Y, N679K, P681H, N764K, D796Y, Q954H, N969K), or the Omicron BA.4/5 variant (alterations: T19I, Δ24-26, A27S, Δ69/70, G142D, V213G, G339D, S371F, S373P, S375F, T376A, D405N, R408S, K417N, N440K, L452R, S477N, T478K, E484A, F486V, Q498R, N501Y, Y505H, D614G, H655Y, N679K, P681H, N764K, D796Y, Q954H, N969K) according to published pseudotyping protocols (*49*).

A diagram of SARS-CoV-2 S glycoprotein alterations is shown in fig. S7a. In brief, HEK293T/17 monolayers (ATCC® CRL-11268) cultured in Dulbecco’s modified Eagle’s medium (DMEM) with GlutaMAX (Gibco) supplemented with 10% heat-inactivated fetal bovine serum (FBS [Sigma-Aldrich]) (referred to as medium) were transfected with Sanger sequencing-verified SARS-CoV-1 or variant-specific SARS-CoV-2 S expression plasmid with Lipofectamine LTX (Life Technologies) following the manufacturer’s instructions. At 24 hours after transfection, the cells were infected at a multiplicity of infection (MOI) of three with VSV-G complemented VSVΔG vector. After incubation for 2 hours at 37°C with 7.5% CO_2_, cells were washed twice with phosphate buffered saline (PBS) before medium supplemented with anti-VSV-G antibody (clone 8G5F11, Kerafast, Inc.) was added to neutralize residual VSV-G-complemented input virus. VSV-SARS-CoV-2-S pseudotype-containing medium was harvested 20 hours after inoculation, passed through a 0.2 μm filter (Nalgene) and stored at -80°C. The pseudovirus batches were titrated on Vero 76 cells (ATCC® CRL-1587) cultured in medium. The relative luciferase units induced by a defined volume of a Wuhan S glycoprotein pseudovirus reference batch previously described in Muik *et al*. (*26*), that corresponds to an infectious titer of 200 transducing units (TU) per mL, was used as a comparator. Input volumes for the SARS-CoV-2 variant pseudovirus batches were calculated to normalize the infectious titer based on the relative luciferase units relative to the reference.

### Pseudovirus neutralization assay

Vero 76 cells were seeded in 96-well white, flat-bottom plates (Thermo Scientific) at 40,000 cells/well in medium 4 hours prior to the assay and cultured at 37°C with 7.5% CO_2_. Each individual serum was serially diluted 2-fold in medium with the first dilution being 1:5 (Omicron-naïve double- and triple BNT162b2 vaccinated; dilution range of 1:5 to 1:5,120) or 1:30 (double- and triple-BNT162b2 vaccinated after subsequent Omicron breakthrough infection; dilution range of 1:30 to 1:30,720). In the case of the SARS-CoV-1 pseudovirus assay, the serum of all individuals was initially diluted 1:5 (dilution range of 1:5 to 1:5,120). VSV-SARS-CoV-2-S/VSV-SARS-CoV-1-S particles were diluted in medium to obtain 200 TU in the assay. Serum dilutions were mixed 1:1 with pseudovirus (n=2 technical replicates per serum per pseudovirus) for 30 min at room temperature before being added to Vero 76 cell monolayers and incubated at 37°C with 7.5% CO_2_ for 24 hours. Supernatants were removed and the cells were lysed with luciferase reagent (Promega). Luminescence was recorded on a CLARIOstar® Plus microplate reader (BMG Labtech), and neutralization titers were calculated as the reciprocal of the highest serum dilution that still resulted in 50% reduction in luminescence. Results were expressed as geometric mean titers (GMT) of duplicates. If no neutralization was observed, an arbitrary titer value of half of the limit of detection (LOD) was reported. Supplementary tables listing the neutralization titers are provided (Tables S4-6 and Table S11).

### Live SARS-CoV-2 neutralization assay

SARS-CoV-2 virus neutralization titers were determined by a microneutralization assay based on cytopathic effect (CPE) at VisMederi S.r.l., Siena, Italy. In brief, heat-inactivated serum samples from individuals were serially diluted 1:2 (starting at 1:10; n=2 technical replicates per serum per virus) and incubated for 1 hour at 37°C with 100 TCID_50_ of live Wuhan-like SARS-CoV-2 virus strain 2019-nCOV/ITALY-INMI1 (GenBank: MT066156), Beta virus strain Human nCoV19 isolate/England ex-SA/HCM002/2021 (alterations: D80A, D215G, Δ242–244, K417N, E484K, N501Y, D614G, A701V), sequence-verified Delta strain isolated from a nasopharyngeal swab (alterations: T19R, G142D, E156G, Δ157/158, L452R, T478K, D614G, P681R, R682Q, D950N) or Omicron BA.1 strain hCoV-19/Belgium/rega-20174/2021 (alterations: A67V, Δ69/70, T95I, G142D, Δ143-145, Δ211, L212I, ins214EPE, G339D, S371L, S373P, S375F, K417N, N440K, G446S, S477N, T478K, E484A, Q493R, G496S, Q498R, N501Y, Y505H, T547K, D614G, H655Y, N679K, P681H, N764K, D796Y, N856K, Q954H, N969K, L981F) to allow any antigen-specific antibodies to bind to the virus. A diagram of S glycoprotein alterations is shown in fig. S7b. The 2019-nCOV/ITALY-INMI1 strain S glycoprotein is identical in sequence to the wild-type SARS-CoV-2 S (Wuhan-Hu-1 isolate). Vero E6 (ATCC® CRL-1586) cell monolayers were inoculated with the serum/virus mix in 96-well plates and incubated for 3 days (2019-nCOV/ITALY-INMI1 strain) or 4 days (Beta, Delta and Omicron BA.1 variant strain) to allow infection by non-neutralized virus. The plates were observed under an inverted light microscope and the wells were scored as positive for SARS-CoV-2 infection (i.e., showing CPE) or negative for SARS-CoV-2 infection (i.e., cells were alive without CPE). The neutralization titer was determined as the reciprocal of the highest serum dilution that protected more than 50% of cells from CPE and reported as GMT of duplicates. If no neutralization was observed, an arbitrary titer value of 5 (half of the LOD) was reported. Supplementary tables listing the neutralization titers are provided (Tables S7 to S9).

### Detection and characterization of SARS-CoV-2-specific B cells with flow cytometry

S glycoprotein/receptor-binding domain (RBD)-specific B cells were detected using recombinant, biotinylated SARS-CoV-2 spike (Acro Biosystems: Wuhan – SPN-C82E9, Alpha – SPN-C82E5, Delta – SPN-C82Ec, Omicron – SPN-C82Ee) and RBD (Acro Biosystems: Wuhan – SPD-B28E9, Alpha – SPD-C82E6, Delta – SPD-C82Ed, Omicron – SPD-C82E4) proteins. Recombinant S and RBD proteins were tetramerized with fluorescently labeled streptavidin (streptavidin-BV421, -AF647, or -PE all from BioLegend; streptavidin-BUV661 from BD Biosciences) in a 4:1 molar ratio for 1 hour at 4°C in the dark. Afterwards samples were spun down for 10 min at 4°C to remove possible precipitates.

For flow cytometric analysis, PBMCs were thawed and 5x10^6^ cells per sample were seeded into 96 U-bottom plates. Cells were blocked for Fc-receptor-binding (Human BD Fc Block, BD Biosciences) and free biotin (D-biotin, Invitrogen, 1 μM) in flow buffer (DPBS (Gibco) supplemented with 2% FBS (Sigma), 2 mM EDTA (Sigma-Aldrich)) for 20 min at 4°C. Cells were washed and labeled with BCR-bait tetramers supplemented with free biotin in flow buffer (D-biotin, Invitrogen, 2 μg/ml) for 1 hour at 4°C in the dark (2 μg/ml for spike and 0.25 μg/ml for RBD proteins). Cells were washed with flow buffer and stained for viability (Fixable Viability Dye eFluor 780, eBioscience) and surface markers (CD3 BUV395 – clone: UCHT1(BD Biosciences, 563546), CD4 BB515 – clone: SK3 (BD Biosciences, 566912), CD185 (CXCR5) PE-Cy7 – clone: RF8B2 (BioLegend, 256924), CD279 (PD-1) BV650 – clone: EH12.1 (BD Biosciences, 564104), CD278 (ICOS) AF700 – clone: C398.4A (BioLegend, 313528), CD19 BV786 clone: SJ25C1 (BD Biosciences, 563326), CD20 BUV496 – clone: 2H7 (BD Biosciences, 749954), CD21 BV786 – clone: B-ly4 (BD Biosciences, 740969), CD27 BUV737 – clone: L128 (BD Biosciences, 612829), CD38 PE-CF594 – clone: HIT2 (BD Biosciences, 562288), CD11c BB700 – clone: S-HCL-3 (BD Biosciences, 746106), CD138 BV711 – clone: MI15 (BD Biosciences, 563184), IgG BUV563 - clone: G18-145 (BD Biosciences, 741396), IgM – clone: G20-127 (BD Biosciences), IgD BV480 – clone: IA6-2 (BD Biosciences, 566146), CD14 APC-H7 – clone: MφP9 (BD Biosciences, 560715, dump channel), CD16 APC-H7 – clone: 3G8 (BD Biosciences, 560180, dump channel)) in flow buffer supplemented with Brilliant Stain Buffer Plus (BD Biosciences, according to the manufacturer’s instructions) for 20 min at 4°C. Samples were washed and fixed with BD^TM^ Stabilizing Fixative (BD Biosciences, according to the manufacturer’s instructions) prior to data acquisition on a BD Symphony A3 flow cytometer. FCS 3.0 files were exported from BD Diva Software and analyzed using FlowJo software (Version 10.7.1.).

### Statistical analysis

The statistical method of aggregation used for the analysis of antibody titers is the geometric mean and for the ratio of SARS-CoV-2 VOC titer and Wuhan titer the geometric mean and the corresponding 95% confidence interval. The use of the geometric mean accounts for the non-normal distribution of antibody titers, which span several orders of magnitude. The Friedman test with Dunn’s correction for multiple comparisons was used to conduct pairwise signed-rank tests of group geometric mean neutralizing antibody titers with a common control group. Flow cytometric frequencies were analyzed with and tables were exported from FlowJo software (Version 10.7.1.). Statistical analysis of cumulative memory B cell frequencies was the mean and standard error of the mean (SEM). Statistical significance was tested for using the nonparametric Friedman test with Dunn’s multiple comparisons correction. All statistical analyses were performed using GraphPad Prism software version 9.

## Supplementary Materials

### Other Supplementary Material for this manuscript includes the following:

MDAR Reproducibility Checklist

## References

[1] D. S. Khoury , D. Cromer , A. Reynaldi , T. E. Schlub , A. K. Wheatley , J. A. Juno , K. Subbarao , S. J. Kent , J. A. Triccas , M. P. Davenport , Neutralizing antibody levels are highly predictive of immune protection from symptomatic SARS-CoV-2 infection. Nat. Med. 27, 1205–1211 (2021). 10.1038/s41591-021-01377-8 34002089

[2] P. B. Gilbert , D. C. Montefiori , A. B. McDermott , Y. Fong , D. Benkeser , W. Deng , H. Zhou , C. R. Houchens , K. Martins , L. Jayashankar , F. Castellino , B. Flach , B. C. Lin , S. O’Connell , C. McDanal , A. Eaton , M. Sarzotti-Kelsoe , Y. Lu , C. Yu , B. Borate , L. W. P. van der Laan , N. S. Hejazi , C. Huynh , J. Miller , H. M. El Sahly , L. R. Baden , M. Baron , L. De La Cruz , C. Gay , S. Kalams , C. F. Kelley , M. P. Andrasik , J. G. Kublin , L. Corey , K. M. Neuzil , L. N. Carpp , R. Pajon , D. Follmann , R. O. Donis , R. A. Koup ; Immune Assays Team§; Moderna, Inc. Team§; Coronavirus Vaccine Prevention Network (CoVPN)/Coronavirus Efficacy (COVE) Team§; United States Government (USG)/CoVPN Biostatistics Team§ , Immune correlates analysis of the mRNA-1273 COVID-19 vaccine efficacy clinical trial. Science 375, 43–50 (2022). 10.1126/science.abm3425 34812653 PMC9017870

[3] K. Röltgen , S. C. A. Nielsen , O. Silva , S. F. Younes , M. Zaslavsky , C. Costales , F. Yang , O. F. Wirz , D. Solis , R. A. Hoh , A. Wang , P. S. Arunachalam , D. Colburg , S. Zhao , E. Haraguchi , A. S. Lee , M. M. Shah , M. Manohar , I. Chang , F. Gao , V. Mallajosyula , C. Li , J. Liu , M. J. Shoura , S. B. Sindher , E. Parsons , N. J. Dashdorj , N. D. Dashdorj , R. Monroe , G. E. Serrano , T. G. Beach , R. S. Chinthrajah , G. W. Charville , J. L. Wilbur , J. N. Wohlstadter , M. M. Davis , B. Pulendran , M. L. Troxell , G. B. Sigal , Y. Natkunam , B. A. Pinsky , K. C. Nadeau , S. D. Boyd , Immune imprinting, breadth of variant recognition, and germinal center response in human SARS-CoV-2 infection and vaccination. Cell 185, 1025–1040.e14 (2022). 10.1016/j.cell.2022.01.018 35148837 PMC8786601

[4] J. P. Evans , C. Zeng , C. Carlin , G. Lozanski , L. J. Saif , E. M. Oltz , R. J. Gumina , S.-L. Liu , Neutralizing antibody responses elicited by SARS-CoV-2 mRNA vaccination wane over time and are boosted by breakthrough infection. Sci. Transl. Med. 14, eabn8057 (2022). 10.1126/scitranslmed.abn8057 35166573 PMC8939766

[5] S. Yamayoshi , A. Yasuhara , M. Ito , O. Akasaka , M. Nakamura , I. Nakachi , M. Koga , K. Mitamura , K. Yagi , K. Maeda , H. Kato , M. Nojima , D. Pattinson , T. Ogura , R. Baba , K. Fujita , H. Nagai , S. Yamamoto , M. Saito , E. Adachi , J. Ochi , S. I. Hattori , T. Suzuki , Y. Miyazato , S. Chiba , M. Okuda , J. Murakami , T. Hamabata , K. Iwatsuki-Horimoto , H. Nakajima , H. Mitsuya , N. Omagari , N. Sugaya , H. Yotsuyanagi , Y. Kawaoka , Antibody titers against SARS-CoV-2 decline, but do not disappear for several months. EClinicalMedicine 32, 100734 (2021). 10.1016/j.eclinm.2021.100734 33589882 PMC7877219

[6] W. N. Chia , F. Zhu , S. W. X. Ong , B. E. Young , S.-W. Fong , N. Le Bert , C. W. Tan , C. Tiu , J. Zhang , S. Y. Tan , S. Pada , Y.-H. Chan , C. Y. L. Tham , K. Kunasegaran , M. I.-C. Chen , J. G. H. Low , Y.-S. Leo , L. Renia , A. Bertoletti , L. F. P. Ng , D. C. Lye , L.-F. Wang , Dynamics of SARS-CoV-2 neutralising antibody responses and duration of immunity: A longitudinal study. Lancet Microbe 2, e240–e249 (2021). 10.1016/S2666-5247(21)00025-2 33778792 PMC7987301

[7] Y. Goldberg , M. Mandel , Y. M. Bar-On , O. Bodenheimer , L. Freedman , E. J. Haas , R. Milo , S. Alroy-Preis , N. Ash , A. Huppert , Waning Immunity after the BNT162b2 Vaccine in Israel. N. Engl. J. Med. 385, e85 (2021). 10.1056/NEJMoa2114228 34706170 PMC8609604

[8] A. R. Falsey , R. W. Frenck Jr ., E. E. Walsh , N. Kitchin , J. Absalon , A. Gurtman , S. Lockhart , R. Bailey , K. A. Swanson , X. Xu , K. Koury , W. Kalina , D. Cooper , J. Zou , X. Xie , H. Xia , Ö. Türeci , E. Lagkadinou , K. R. Tompkins , P.-Y. Shi , K. U. Jansen , U. Şahin , P. R. Dormitzer , W. C. Gruber , SARS-CoV-2 Neutralization with BNT162b2 Vaccine Dose 3. N. Engl. J. Med. 385, 1627–1629 (2021). 10.1056/NEJMc2113468 34525276 PMC8461567

[9] A. Choi , M. Koch , K. Wu , L. Chu , L. Ma , A. Hill , N. Nunna , W. Huang , J. Oestreicher , T. Colpitts , H. Bennett , H. Legault , Y. Paila , B. Nestorova , B. Ding , D. Montefiori , R. Pajon , J. M. Miller , B. Leav , A. Carfi , R. McPhee , D. K. Edwards , Safety and immunogenicity of SARS-CoV-2 variant mRNA vaccine boosters in healthy adults: An interim analysis. Nat. Med. 27, 2025–2031 (2021). 10.1038/s41591-021-01527-y 34526698 PMC8604720

[10] N. Andrews , J. Stowe , F. Kirsebom , S. Toffa , R. Sachdeva , C. Gower , M. Ramsay , J. Lopez Bernal , Effectiveness of COVID-19 booster vaccines against COVID-19-related symptoms, hospitalization and death in England. Nat. Med. 28, 831–837 (2022). 10.1038/s41591-022-01699-1 35045566 PMC9018410

[11] WHO Technical Advisory Group on COVID-19 Vaccine Composition (TAG-CO-VAC), *Interim statement on COVID-19 vaccines in the context of the circulation of the Omicron SARS-CoV-2 variant* *.* (2022) (available at https://www.who.int/news/item/08-03-2022-interim-statement-on-covid-19-vaccines-in-the-context-of-the-circulation-of-the-Omicron-sars-cov-2-variant-from-the-who-technical-advisory-group-on-covid-19-vaccine-composition-(tag-co-vac)-08-march-2022).

[12] W. E. Purtha , T. F. Tedder , S. Johnson , D. Bhattacharya , M. S. Diamond , Memory B cells, but not long-lived plasma cells, possess antigen specificities for viral escape mutants. J. Exp. Med. 208, 2599–2606 (2011). 10.1084/jem.20110740 22162833 PMC3244041

[13] Y. Adachi , T. Onodera , Y. Yamada , R. Daio , M. Tsuiji , T. Inoue , K. Kobayashi , T. Kurosaki , M. Ato , Y. Takahashi , Distinct germinal center selection at local sites shapes memory B cell response to viral escape. J. Exp. Med. 212, 1709–1723 (2015). 10.1084/jem.20142284 26324444 PMC4577849

[14] E. Mathieu , H. Ritchie , E. Ortiz-Ospina , M. Roser , J. Hasell , C. Appel , C. Giattino , L. Rodés-Guirao , A global database of COVID-19 vaccinations. Nat. Hum. Behav. 5, 947–953 (2021). 10.1038/s41562-021-01122-8 33972767

[15] WHO Headquarters (HQ), WHO Health Emergencies Programme, *Enhancing response to Omicron SARS-CoV-2 variant: Technical brief and priority actions for Member States* (2022).

[16] L. Premkumar , B. Segovia-Chumbez , R. Jadi , D. R. Martinez , R. Raut , A. Markmann , C. Cornaby , L. Bartelt , S. Weiss , Y. Park , C. E. Edwards , E. Weimer , E. M. Scherer , N. Rouphael , S. Edupuganti , D. Weiskopf , L. V. Tse , Y. J. Hou , D. Margolis , A. Sette , M. H. Collins , J. Schmitz , R. S. Baric , A. M. de Silva , The receptor binding domain of the viral spike protein is an immunodominant and highly specific target of antibodies in SARS-CoV-2 patients. Sci. Immunol. 5, eabc8413 (2020). 10.1126/sciimmunol.abc8413 32527802 PMC7292505

[17] W. T. Harvey , A. M. Carabelli , B. Jackson , R. K. Gupta , E. C. Thomson , E. M. Harrison , C. Ludden , R. Reeve , A. Rambaut , S. J. Peacock , D. L. Robertson ; COVID-19 Genomics UK (COG-UK) Consortium , SARS-CoV-2 variants, spike mutations and immune escape. Nat. Rev. Microbiol. 19, 409–424 (2021). 10.1038/s41579-021-00573-0 34075212 PMC8167834

[18] M. Hoffmann , N. Krüger , S. Schulz , A. Cossmann , C. Rocha , A. Kempf , I. Nehlmeier , L. Graichen , A.-S. Moldenhauer , M. S. Winkler , M. Lier , A. Dopfer-Jablonka , H.-M. Jäck , G. M. N. Behrens , S. Pöhlmann , The Omicron variant is highly resistant against antibody-mediated neutralization: Implications for control of the COVID-19 pandemic. Cell 185, 447–456.e11 (2022). 10.1016/j.cell.2021.12.032 35026151 PMC8702401

[19] W. Dejnirattisai , J. Huo , D. Zhou , J. Zahradník , P. Supasa , C. Liu , H. M. E. Duyvesteyn , H. M. Ginn , A. J. Mentzer , A. Tuekprakhon , R. Nutalai , B. Wang , A. Dijokaite , S. Khan , O. Avinoam , M. Bahar , D. Skelly , S. Adele , S. A. Johnson , A. Amini , T. G. Ritter , C. Mason , C. Dold , D. Pan , S. Assadi , A. Bellass , N. Omo-Dare , D. Koeckerling , A. Flaxman , D. Jenkin , P. K. Aley , M. Voysey , S. A. Costa Clemens , F. G. Naveca , V. Nascimento , F. Nascimento , C. Fernandes da Costa , P. C. Resende , A. Pauvolid-Correa , M. M. Siqueira , V. Baillie , N. Serafin , G. Kwatra , K. Da Silva , S. A. Madhi , M. C. Nunes , T. Malik , P. J. M. Openshaw , J. K. Baillie , M. G. Semple , A. R. Townsend , K. A. Huang , T. K. Tan , M. W. Carroll , P. Klenerman , E. Barnes , S. J. Dunachie , B. Constantinides , H. Webster , D. Crook , A. J. Pollard , T. Lambe , N. G. Paterson , M. A. Williams , D. R. Hall , E. E. Fry , J. Mongkolsapaya , J. Ren , G. Schreiber , D. I. Stuart , G. R. Screaton ; OPTIC Consortium; ISARIC4C Consortium , SARS-CoV-2 Omicron-B.1.1.529 leads to widespread escape from neutralizing antibody responses. Cell 185, 467–484.e15 (2022). 10.1016/j.cell.2021.12.046 35081335 PMC8723827

[20] Y. Cao , J. Wang , F. Jian , T. Xiao , W. Song , A. Yisimayi , W. Huang , Q. Li , P. Wang , R. An , J. Wang , Y. Wang , X. Niu , S. Yang , H. Liang , H. Sun , T. Li , Y. Yu , Q. Cui , S. Liu , X. Yang , S. Du , Z. Zhang , X. Hao , F. Shao , R. Jin , X. Wang , J. Xiao , Y. Wang , X. S. Xie , Omicron escapes the majority of existing SARS-CoV-2 neutralizing antibodies. Nature 602, 657–663 (2022). 10.1038/s41586-021-04385-3 35016194 PMC8866119

[21] S. Mallapaty , COVID-19: How Omicron overtook Delta in three charts. Nature (2022). 10.1038/d41586-022-00632-3 35246640

[22] European Centre for Disease Prevention and Control, *Assessment of the further spread and potential impact of the SARS-CoV-2 Omicron variant of concern in the EU/EEA, 19th update* (2022) (available at https://www.ecdc.europa.eu/sites/default/files/documents/RRA-19-update-27-jan-2022.pdf).

[23] Euopean Centre for Disease Prevention and Control, *Epidemiological update: SARS-CoV-2 Omicron sub-lineages BA.4 and BA.5* (2022) (available at https://www.ecdc.europa.eu/en/news-events/epidemiological-update-sars-cov-2-omicron-sub-lineages-ba4-and-ba5).

[24] European Centre for Disease Prevention and Control, *Weekly COVID-19 country overview - Country overview report: week 19 2022* (2022) (available at https://www.ecdc.europa.eu/en/covid-19/country-overviews).

[25] A. Muik , B. G. Lui , A.-K. Wallisch , M. Bacher , J. Mühl , J. Reinholz , O. Ozhelvaci , N. Beckmann , R. C. Güimil Garcia , A. Poran , S. Shpyro , A. Finlayson , H. Cai , Q. Yang , K. A. Swanson , Ö. Türeci , U. Şahin , Neutralization of SARS-CoV-2 Omicron by BNT162b2 mRNA vaccine-elicited human sera. Science 375, 678–680 (2022). 10.1126/science.abn7591 35040667 PMC9836206

[26] A. Muik , A.-K. Wallisch , B. Sänger , K. A. Swanson , J. Mühl , W. Chen , H. Cai , D. Maurus , R. Sarkar , Ö. Türeci , P. R. Dormitzer , U. Şahin , Neutralization of SARS-CoV-2 lineage B.1.1.7 pseudovirus by BNT162b2 vaccine-elicited human sera. Science 371, 1152–1153 (2021). 10.1126/science.abg6105 33514629 PMC7971771

[27] C.-W. Tan , W.-N. Chia , B. E. Young , F. Zhu , B.-L. Lim , W.-R. Sia , T.-L. Thein , M. I.-C. Chen , Y.-S. Leo , D. C. Lye , L.-F. Wang , Pan-Sarbecovirus Neutralizing Antibodies in BNT162b2-Immunized SARS-CoV-1 Survivors. N. Engl. J. Med. 385, 1401–1406 (2021). 10.1056/NEJMoa2108453 34407341 PMC8422514

[28] J. Liu , Y. Liu , H. Xia , J. Zou , S. C. Weaver , K. A. Swanson , H. Cai , M. Cutler , D. Cooper , A. Muik , K. U. Jansen , U. Sahin , X. Xie , P. R. Dormitzer , P.-Y. Shi , BNT162b2-elicited neutralization of B.1.617 and other SARS-CoV-2 variants. Nature 596, 273–275 (2021). 10.1038/s41586-021-03693-y 34111888

[29] U. Sahin , A. Muik , I. Vogler , E. Derhovanessian , L. M. Kranz , M. Vormehr , J. Quandt , N. Bidmon , A. Ulges , A. Baum , K. E. Pascal , D. Maurus , S. Brachtendorf , V. Lörks , J. Sikorski , P. Koch , R. Hilker , D. Becker , A.-K. Eller , J. Grützner , M. Tonigold , C. Boesler , C. Rosenbaum , L. Heesen , M.-C. Kühnle , A. Poran , J. Z. Dong , U. Luxemburger , A. Kemmer-Brück , D. Langer , M. Bexon , S. Bolte , T. Palanche , A. Schultz , S. Baumann , A. J. Mahiny , G. Boros , J. Reinholz , G. T. Szabó , K. Karikó , P.-Y. Shi , C. Fontes-Garfias , J. L. Perez , M. Cutler , D. Cooper , C. A. Kyratsous , P. R. Dormitzer , K. U. Jansen , Ö. Türeci , BNT162b2 vaccine induces neutralizing antibodies and poly-specific T cells in humans. Nature 595, 572–577 (2021). 10.1038/s41586-021-03653-6 34044428

[30] R. Kotaki , Y. Adachi , S. Moriyama , T. Onodera , S. Fukushi , T. Nagakura , K. Tonouchi , K. Terahara , L. Sun , T. Takano , A. Nishiyama , M. Shinkai , K. Oba , F. Nakamura-Uchiyama , H. Shimizu , T. Suzuki , T. Matsumura , M. Isogawa , Y. Takahashi , SARS-CoV-2 Omicron-neutralizing memory B cells are elicited by two doses of BNT162b2 mRNA vaccine. Sci. Immunol. 7, eabn8590 (2022). 10.1126/sciimmunol.abn8590 35113654 PMC8939773

[31] T. N. Starr , A. J. Greaney , A. S. Dingens , J. D. Bloom , Complete map of SARS-CoV-2 RBD mutations that escape the monoclonal antibody LY-CoV555 and its cocktail with LY-CoV016. Cell Rep. Med. 2, 100255 (2021). 10.1016/j.xcrm.2021.100255 33842902 PMC8020059

[32] Y. Wang , C. Liu , C. Zhang , Y. Wang , Q. Hong , S. Xu , Z. Li , Y. Yang , Z. Huang , Y. Cong , Structural basis for SARS-CoV-2 Delta variant recognition of ACE2 receptor and broadly neutralizing antibodies. Nat. Commun. 13, 871 (2022). 10.1038/s41467-022-28528-w 35169135 PMC8847413

[33] Y. Cao *et al*., BA.2.12.1, BA.4 and BA.5 escape antibodies elicited by Omicron infection. *bioRxiv* *: * *the preprint server for biology* (2022). 10.1101/2022.04.30.489997 PMC9385493 35714668

[34] G. Cerutti , Y. Guo , L. Liu , L. Liu , Z. Zhang , Y. Luo , Y. Huang , H. H. Wang , D. D. Ho , Z. Sheng , L. Shapiro , Cryo-EM structure of the SARS-CoV-2 Omicron spike. Cell Rep. 38, 110428 (2022). 10.1016/j.celrep.2022.110428 35172173 PMC8818377

[35] S. Cele , L. Jackson , D. S. Khoury , K. Khan , T. Moyo-Gwete , H. Tegally , J. E. San , D. Cromer , C. Scheepers , D. G. Amoako , F. Karim , M. Bernstein , G. Lustig , D. Archary , M. Smith , Y. Ganga , Z. Jule , K. Reedoy , S.-H. Hwa , J. Giandhari , J. M. Blackburn , B. I. Gosnell , S. S. Abdool Karim , W. Hanekom , A. von Gottberg , J. N. Bhiman , R. J. Lessells , M. S. Moosa , M. P. Davenport , T. de Oliveira , P. L. Moore , A. Sigal ; NGS-SA; COMMIT-KZN Team , Omicron extensively but incompletely escapes Pfizer BNT162b2 neutralization. Nature 602, 654–656 (2022). 10.1038/s41586-021-04387-1 35016196 PMC8866126

[36] A. C. Walls , K. R. Sprouse , J. E. Bowen , A. Joshi , N. Franko , M. J. Navarro , C. Stewart , E. Cameroni , M. McCallum , E. A. Goecker , E. J. Degli-Angeli , J. Logue , A. Greninger , D. Corti , H. Y. Chu , D. Veesler , SARS-CoV-2 breakthrough infections elicit potent, broad, and durable neutralizing antibody responses. Cell 185, 872–880.e3 (2022). 10.1016/j.cell.2022.01.011 35123650 PMC8769922

[37] R. Nutalai , D. Zhou , A. Tuekprakhon , H. M. Ginn , P. Supasa , C. Liu , J. Huo , A. J. Mentzer , H. M. E. Duyvesteyn , A. Dijokaite-Guraliuc , D. Skelly , T. G. Ritter , A. Amini , S. Bibi , S. Adele , S. A. Johnson , B. Constantinides , H. Webster , N. Temperton , P. Klenerman , E. Barnes , S. J. Dunachie , D. Crook , A. J. Pollard , T. Lambe , P. Goulder , N. G. Paterson , M. A. Williams , D. R. Hall , J. Mongkolsapaya , E. E. Fry , W. Dejnirattisai , J. Ren , D. I. Stuart , G. R. Screaton , C. Conlon , A. Deeks , J. Frater , L. Frending , S. Gardiner , A. Jämsén , K. Jeffery , T. Malone , E. Phillips , L. Rothwell , L. Stafford , Potent cross-reactive antibodies following Omicron breakthrough in vaccinees. Cell (2022). 10.1016/j.cell.2022.05.014 PMC9120130 35662412

[38] L. Liu , P. Wang , M. S. Nair , J. Yu , M. Rapp , Q. Wang , Y. Luo , J. F.-W. Chan , V. Sahi , A. Figueroa , X. V. Guo , G. Cerutti , J. Bimela , J. Gorman , T. Zhou , Z. Chen , K.-Y. Yuen , P. D. Kwong , J. G. Sodroski , M. T. Yin , Z. Sheng , Y. Huang , L. Shapiro , D. D. Ho , Potent neutralizing antibodies against multiple epitopes on SARS-CoV-2 spike. Nature 584, 450–456 (2020). 10.1038/s41586-020-2571-7 32698192

[39] D. Pinto , Y.-J. Park , M. Beltramello , A. C. Walls , M. A. Tortorici , S. Bianchi , S. Jaconi , K. Culap , F. Zatta , A. De Marco , A. Peter , B. Guarino , R. Spreafico , E. Cameroni , J. B. Case , R. E. Chen , C. Havenar-Daughton , G. Snell , A. Telenti , H. W. Virgin , A. Lanzavecchia , M. S. Diamond , K. Fink , D. Veesler , D. Corti , Cross-neutralization of SARS-CoV-2 by a human monoclonal SARS-CoV antibody. Nature 583, 290–295 (2020). 10.1038/s41586-020-2349-y 32422645

[40] M. M. Sauer , M. A. Tortorici , Y.-J. Park , A. C. Walls , L. Homad , O. J. Acton , J. E. Bowen , C. Wang , X. Xiong , W. de van der Schueren , J. Quispe , B. G. Hoffstrom , B.-J. Bosch , A. T. McGuire , D. Veesler , Structural basis for broad coronavirus neutralization. Nat. Struct. Mol. Biol. 28, 478–486 (2021). 10.1038/s41594-021-00596-4 33981021

[41] T. Li , W. Xue , Q. Zheng , S. Song , C. Yang , H. Xiong , S. Zhang , M. Hong , Y. Zhang , H. Yu , Y. Zhang , H. Sun , Y. Huang , T. Deng , X. Chi , J. Li , S. Wang , L. Zhou , T. Chen , Y. Wang , T. Cheng , T. Zhang , Q. Yuan , Q. Zhao , J. Zhang , J. S. McLellan , Z. H. Zhou , Z. Zhang , S. Li , Y. Gu , N. Xia , Cross-neutralizing antibodies bind a SARS-CoV-2 cryptic site and resist circulating variants. Nat. Commun. 12, 5652 (2021). 10.1038/s41467-021-25997-3 34580306 PMC8476643

[42] M. Yuan , N. C. Wu , X. Zhu , C. D. Lee , R. T. Y. So , H. Lv , C. K. P. Mok , I. A. Wilson , A highly conserved cryptic epitope in the receptor binding domains of SARS-CoV-2 and SARS-CoV. Science 368, 630–633 (2020). 10.1126/science.abb7269 32245784 PMC7164391

[43] D. Pinto , M. M. Sauer , N. Czudnochowski , J. S. Low , M. A. Tortorici , M. P. Housley , J. Noack , A. C. Walls , J. E. Bowen , B. Guarino , L. E. Rosen , J. di Iulio , J. Jerak , H. Kaiser , S. Islam , S. Jaconi , N. Sprugasci , K. Culap , R. Abdelnabi , C. Foo , L. Coelmont , I. Bartha , S. Bianchi , C. Silacci-Fregni , J. Bassi , R. Marzi , E. Vetti , A. Cassotta , A. Ceschi , P. Ferrari , P. E. Cippà , O. Giannini , S. Ceruti , C. Garzoni , A. Riva , F. Benigni , E. Cameroni , L. Piccoli , M. S. Pizzuto , M. Smithey , D. Hong , A. Telenti , F. A. Lempp , J. Neyts , C. Havenar-Daughton , A. Lanzavecchia , F. Sallusto , G. Snell , H. W. Virgin , M. Beltramello , D. Corti , D. Veesler , Broad betacoronavirus neutralization by a stem helix-specific human antibody. Science 373, 1109–1116 (2021). 10.1126/science.abj3321 34344823 PMC9268357

[44] W. Li , Y. Chen , J. Prévost , I. Ullah , M. Lu , S. Y. Gong , A. Tauzin , R. Gasser , D. Vézina , S. P. Anand , G. Goyette , D. Chaterjee , S. Ding , W. D. Tolbert , M. W. Grunst , Y. Bo , S. Zhang , J. Richard , F. Zhou , R. K. Huang , L. Esser , A. Zeher , M. Côté , P. Kumar , J. Sodroski , D. Xia , P. D. Uchil , M. Pazgier , A. Finzi , W. Mothes , Structural basis and mode of action for two broadly neutralizing antibodies against SARS-CoV-2 emerging variants of concern. Cell Rep. 38, 110210 (2022). 10.1016/j.celrep.2021.110210 34971573 PMC8673750

[45] N. K. Hurlburt , L. J. Homad , I. Sinha , M. F. Jennewein , A. J. MacCamy , Y.-H. Wan , J. Boonyaratanakornkit , A. M. Sholukh , A. M. Jackson , P. Zhou , D. R. Burton , R. Andrabi , G. Ozorowski , A. B. Ward , L. Stamatatos , M. Pancera , A. T. McGuire , Structural definition of a pan-sarbecovirus neutralizing epitope on the spike S2 subunit. Commun. Biol. 5, 342 (2022). 10.1038/s42003-022-03262-7 35411021 PMC9001700

[46] K. Khan *et al.* *, * *Omicron sub-lineages BA.4/BA.5 escape BA.1 infection elicited neutralizing immunity* (2022).

[47] F. Muecksch , Z. Wang , A. Cho , C. Gaebler , T. Ben Tanfous , J. DaSilva , E. Bednarski , V. Ramos , S. Zong , B. Johnson , R. Raspe , D. Schaefer-Babajew , I. Shimeliovich , M. Daga , K.-H. Yao , F. Schmidt , K. G. Millard , M. Turroja , M. Jankovic , T. Y. Oliveira , A. Gazumyan , M. Caskey , T. Hatziioannou , P. D. Bieniasz , M. C. Nussenzweig , Increased Memory B Cell Potency and Breadth After a SARS-CoV-2 mRNA Boost. Nature (2022). 10.1038/s41586-022-04778-y 35447027 PMC9259484

[48] W. Kim , J. Q. Zhou , S. C. Horvath , A. J. Schmitz , A. J. Sturtz , T. Lei , Z. Liu , E. Kalaidina , M. Thapa , W. B. Alsoussi , A. Haile , M. K. Klebert , T. Suessen , L. Parra-Rodriguez , P. A. Mudd , S. P. J. Whelan , W. D. Middleton , S. A. Teefey , I. Pusic , J. A. O’Halloran , R. M. Presti , J. S. Turner , A. H. Ellebedy , Germinal centre-driven maturation of B cell response to mRNA vaccination. Nature 604, 141–145 (2022). 10.1038/s41586-022-04527-1 35168246 PMC9204750

[49] M. Berger Rentsch , G. Zimmer , A vesicular stomatitis virus replicon-based bioassay for the rapid and sensitive determination of multi-species type I interferon. PLOS ONE 6, e25858 (2011). 10.1371/journal.pone.0025858 21998709 PMC3187809

