# Supplementary material for: Omicron BA.1 breakthrough infection drives cross-variant neutralization and memory B cell formation against conserved epitopes
Source: Sci Immunol. 2022 Jun 2:eabq2427. doi: 10.1126/sciimmunol.abq2427 (PMC9162083; doi:10.1126/sciimmunol.abq2427)
Supplement: Supplementary file 1 — Figs. S1 to S7 Tables S1 to S13 [file sciimmunol.abq2427_sm.pdf]

Science Immunology
AAAS

Supplementary Materials for

Omicron BA.1 breakthrough infection drives cross-variant neutralization and memory B cell formation against conserved epitopes

Jasmin Quandt *et al.*

Corresponding author: Ugur Sahin, Ugur.Sahin@biontech.de

DOI: 10.1126/sciimmunol.abq2427

The PDF file includes:

Figs. S1 to S7
Tables S1 to S13

Other Supplementary Material for this manuscript includes the following:

MDAR Reproducibility Checklist

Omicron BA.1 breakthrough infection drives cross-variant neutralization and memory B cell formation against conserved epitopes

Jasmin Quandt, Alexander Muik, Nadine Salisch, Bonny Gaby Lui, Sebastian Lutz, Kimberly Krüger, Ann-Kathrin Wallisch, Petra Adams-Quack, Maren Bacher, Andrew Finlayson, Orkun Ozhelvaci, Isabel Vogler, Katharina Grikscheit, Sebastian Hoehl, Udo Goetsch, Sandra Ciesek, Özlem Türeci, Ugur Sahin

Correspondence to: Ugur.Sahin@biontech.de

Supplementary Materials:

Figs. S1-S7

Tables S1-S13

Reproducibility Checklist

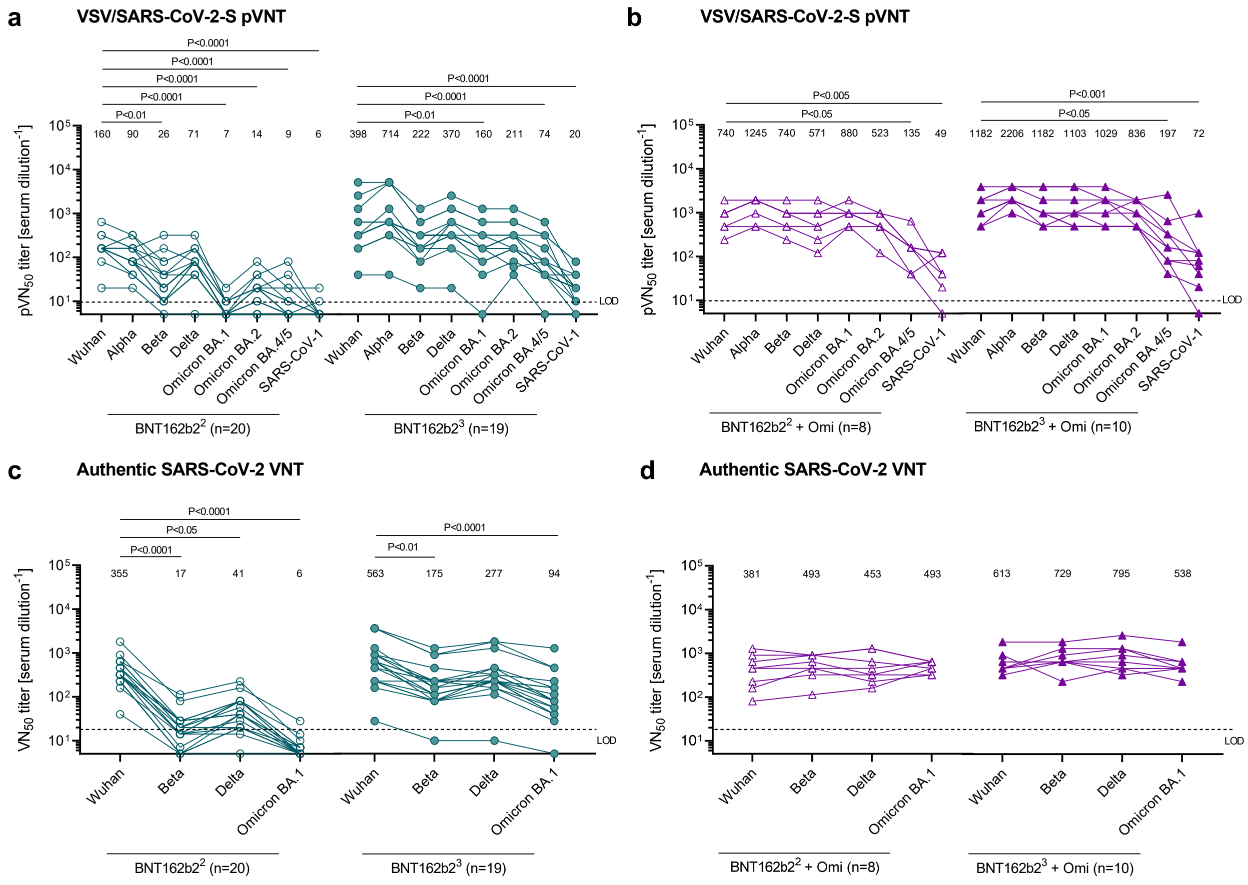

Fig. S1. Individually plotted 50% pseudovirus and live SARS-CoV-2 virus neutralization titers (pVN_{50} and VN_{50}) against SARS-CoV-2 Wuhan, variants of concern (VOCs) and SARS-CoV-1.

Individual line plots from the dataset shown in Fig. 2a, b. pVN_{50} GMTs against Wuhan, VOC and SARS-CoV-1 pseudovirus are shown for (**a**) Omicron-naïve individuals double- ($BNT162b2^2$: green, open circles) and triple-vaccinated with BNT162b2 ($BNT162b2^3$: green, closed circles), and (**b**) Omicron BA.1 breakthrough infected individuals double- (BNT162b22 + Omi: purple, open triangles) and triple-vaccinated with BNT162b2 ($BNT162b2^3$ + Omi: purple, closed triangles) prior to infection. VN_{50} GMTs against Wuhan and VOC are shown for (**c**) Omicron-naïve individuals double- ($BNT162b2^2$: green, open circles) and triple-vaccinated with

BNT162b2 (BNT162b2^3: green, closed circles), and (**d**) Omicron BA.1 breakthrough infected individuals double- (BNT162b2^2 + Omi: purple, open triangles) and triple-vaccinated with BNT162b2 (BNT162b2^3 + Omi: purple, closed triangles) prior to infection. Serum was tested in duplicate. For titer values below the limit of detection (LOD), LOD/2 values were plotted. Values above line plots represent group GMTs. The nonparametric Friedman test with Dunn's multiple comparisons correction was used to compare Wuhan neutralizing group GMTs with titers against the indicated variants and SARS-CoV-1. Multiplicity-adjusted p values are shown.

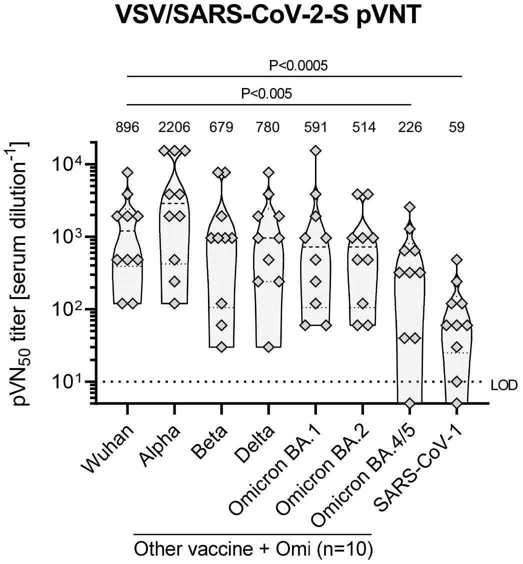

Fig. S2. Omicron BA.1 breakthrough infection of individuals vaccinated with other approved COVID-19 vaccines or mixed regimens results in immune sera that broadly neutralize Omicron BA.1, BA.2 and other VOCs, but not BA.4 and BA.5.

Serum was drawn from 10 individuals vaccinated with other approved COVID-19 vaccines or mixed regimens at a median 43 days after infection (grey diamonds). Serum was tested in duplicate; individual 50% pseudovirus neutralization (pVN50) geometric mean titers (GMTs) against SARS-CoV-2 Wuhan, Alpha, Beta, Delta and Omicron BA.1, BA.2 and BA.4/5 variants, plus SARS-CoV-1 were plotted. For titer values below the limit of detection (LOD), LOD/2 values were plotted. Values above violin plots represent group GMTs. The nonparametric Friedman test with Dunn's multiple comparisons correction was used to compare Wuhan neutralizing group GMTs with titers against the indicated variants and SARS-CoV-1. Multiplicity-adjusted p values are shown.

Fig. S3

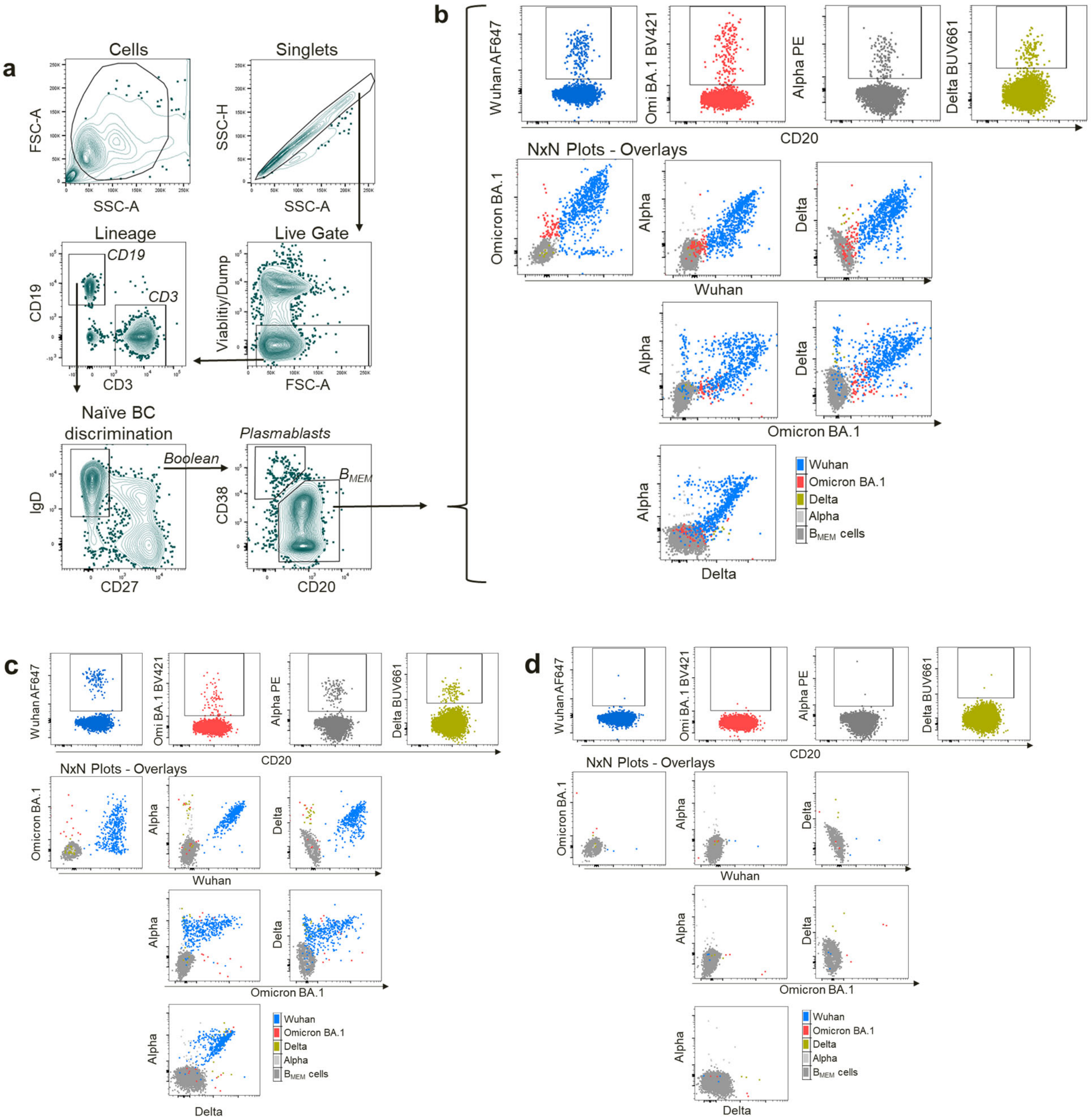
a
Cells
FSC-A
SSC-A
Singlets
SSC-H
SSC-A
Lineage
CD19
CD3
Live Gate
Viability/Dump
FSC-A
Naïve BC discrimination
IgD
CD27
Boolean
Plasmablasts
B_{MEM}
CD38
CD20
b
Wuhan AF647
Omi BA.1 BV421
Alpha PE
Delta BUV661
CD20
NxN Plots - Overlays
Omicron BA.1
Alpha
Delta
Wuhan
Omicron BA.1
Delta
Wuhan
Omicron BA.1
Delta
Alpha
B_{MEM} cells
c
NxN Plots - Overlays
d
NxN Plots - Overlays

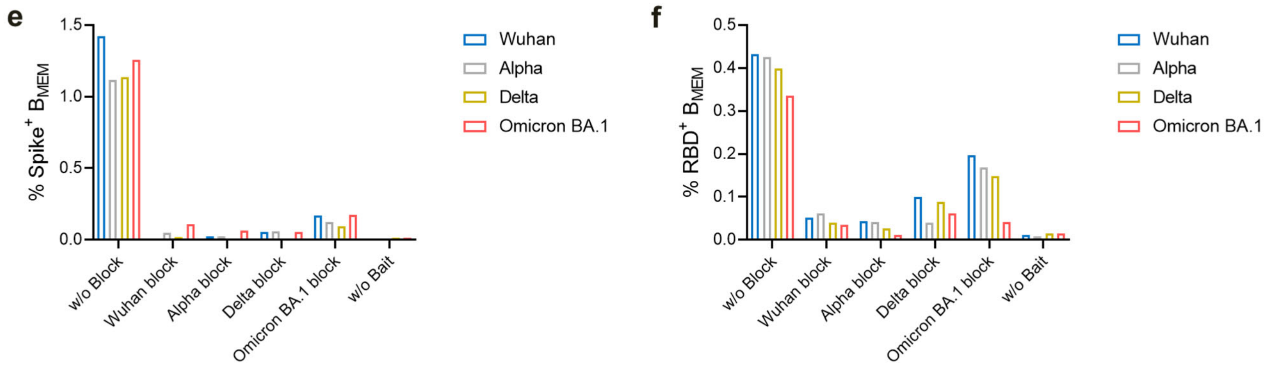

Fig. S3. Flow cytometric gating strategy for B cell phenotypic analysis and assay controls.

Representative flow plots of a triple BNT162b2 vaccinated and Omicron BA.1 convalescent subject analyzed for binding of full-length SARS-COV-2 Wuhan spike (S) glycoprotein and its variants (also referred to as B-cell baits). (**a**) Debris and doublets were discriminated via forward scatter (FSC)/side scatter (SSC). Dead cells and monocytes (CD14, CD16 – Viability/Dump channel) were then excluded. CD19 positive B cells were analyzed for IgD and CD27 expression: thereby, naïve B cells were discriminated as IgD^+ cells with the Boolean 'make non-gate' function. Within non-naïve B cells, plasmablasts ($CD38^{high}$ $CD20^{low}$) and memory B cells (B_{MEMS} $CD38^{int/low}CD20^{high}$) were distinguished. (**b**) B_{MEM} were analyzed for B-cell bait binding. SARS-CoV-2 S reactivities were assessed by gating on each S/RBD variant tested by plotting against the CD20 signal. Bait gates were overlayed onto total B_{MEM} cells and displayed as NxN-plots for the four bait channels. (**c**) The same analysis is shown for a sample from the same donor stained with Wuhan RBD and the other three variants tested as well as (**d**) the negative staining control stained with the four different streptavidin-fluorophore conjugates (StrepAV-BV421, StrepAV-BUV661, StrepAV-AF647, StrepAV-PE) used for tetramerization without addition of the bait proteins. To further ensure specific binding, samples from a BNT162b2-vaccinated subject were pre-incubated with non-labeled S glycoprotein (**e**) and RBD (**f**) prior to bait

staining. This shows that pre-incubation with non-labeled S glycoprotein and RBD prevents fluorescently labeled bait proteins from binding.

Fig. S4

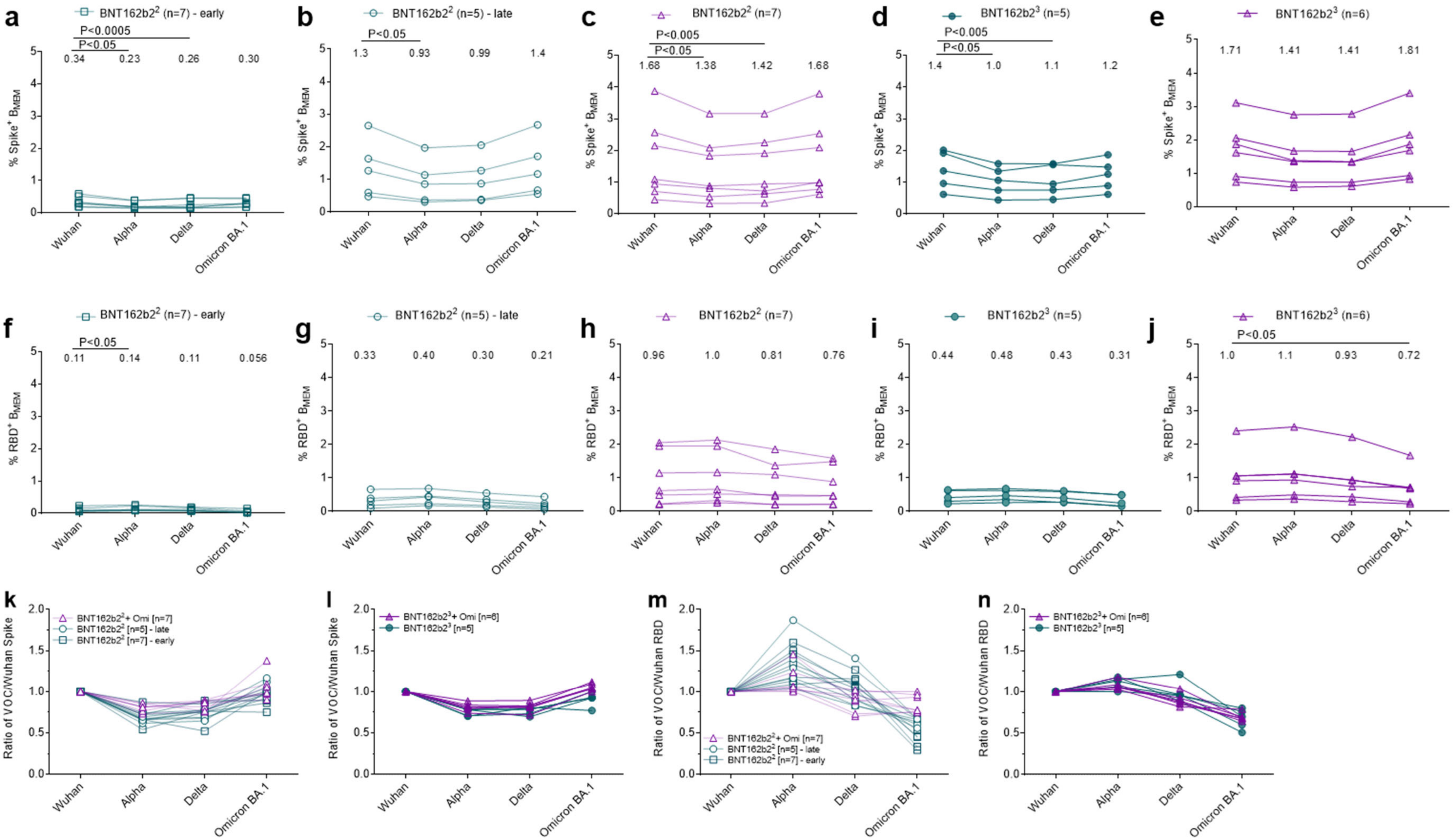
a
BNT162b2² (n=7) - early
P<0.0005
P<0.05
0.34 0.23 0.26 0.30
% Spike⁺ B_MEM
Wuhan
Alpha
Delta
Omicron BA.1
b
BNT162b2² (n=5) - late
P<0.05
1.3 0.93 0.99 1.4
c
BNT162b2² (n=7)
P<0.005
P<0.05
1.68 1.38 1.42 1.68
d
BNT162b2³ (n=5)
P<0.005
P<0.05
1.4 1.0 1.1 1.2
e
BNT162b2³ (n=6)
1.71 1.41 1.41 1.81
f
BNT162b2² (n=7) - early
P<0.05
0.11 0.14 0.11 0.056
% RBD⁺ B_MEM
g
BNT162b2² (n=5) - late
0.33 0.40 0.30 0.21
h
BNT162b2² (n=7)
0.96 1.0 0.81 0.76
i
BNT162b2³ (n=5)
0.44 0.48 0.43 0.31
j
BNT162b2³ (n=6)
P<0.05
1.0 1.1 0.93 0.72
k
Ratio of VOC/Wuhan Spike
BNT162b2²+ Omi [n=7]
BNT162b2² [n=5] - late
BNT162b2² [n=7] - early
l
BNT162b2³+ Omi [n=6]
BNT162b2³ [n=5]
m
Ratio of VOC/Wuhan RBD
BNT162b2²+ Omi [n=7]
BNT162b2² [n=5] - late
BNT162b2² [n=7] - early
n
BNT162b2³+ Omi [n=6]
BNT162b2³ [n=5]

Fig. S4. Omicron BA.1 breakthrough infection of BNT162b2 double- and of triple-vaccinated individuals primarily boosts B_{MEM} against conserved epitopes shared broadly between S proteins of Wuhan and other VOCs rather than strictly Omicron BA.1 spike-specific epitopes.

PBMC samples from double (BNT162b2^2) and triple (BNT162b2^3) BNT162b2 vaccinated individuals who did (green) or did not (purple) experience an Omicron BA.1 breakthrough infection (+Omi) were analyzed via flow cytometry for antigen-specific memory B cell (B_{MEM} – $CD3^-CD19^+CD20^+IgD^-CD38^{int/low}$) frequencies via B-cell bait staining. Frequencies of Wuhan, Alpha, Delta, and Omicron BA.1 S glycoprotein specific B_{MEM} (**a-e**), and Wuhan and variants RBD-specific B_{MEM} cells (**f-j**) for the five different groups of individuals are shown. Each line represents one individual donor. Variant-specific B_{MEM} frequencies were normalized to Wuhan frequencies for full-length S (**k, l**) and RBD (**m, n**) binding for Omicron-naïve and -experienced individuals that received two doses of BNT162b2 (**k, m**) and triple-dosed individuals (**l, n**). Each line represents one individual donor. Mean values are indicated. n = number of individuals per group. The nonparametric Friedman test with Dunn’s multiple comparisons correction was used to compare frequencies of Wuhan S- and RBD-specific B_{MEM} cells to the variants. VOC, variant of concern; FLS, full-length S glycoprotein.

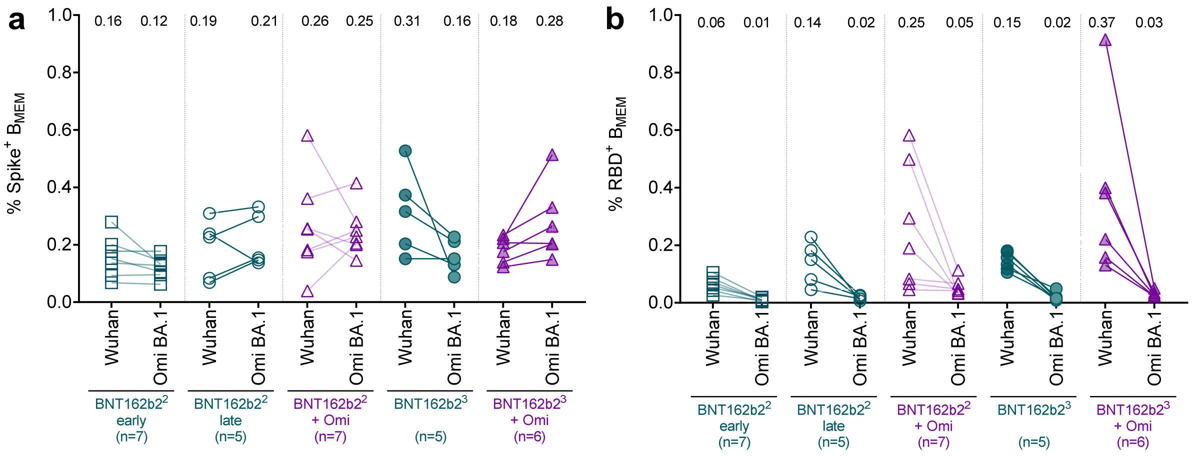

Fig. S5. Omicron BA.1 breakthrough infection of BNT162b2 double- and triple-vaccinated individuals primarily boosts B_{MEM} against conserved epitopes shared broadly between S glycoproteins of Wuhan and other VOCs rather than strictly Omicron BA.1 S-specific epitopes.

PBMC samples from double-vaccinated individuals (BNT162b2^2) at 22 days after the second dose (green, open squares) and 5 months after the second dose (green, open circles), from triple-vaccinated individuals (BNT162b2^3) at 84 days after the third dose (green, closed circles), from double-vaccinated individuals with Omicron BA.1 breakthrough infection (BNT162b2^2 + Omi) at 46 days post-infection (purple, open triangles), and from triple-vaccinated individuals with Omicron BA.1 breakthrough infection (BNT162b2^3 + Omi) at 44 days post-infection (purple, closed triangle) were analyzed via flow cytometry for SARS-CoV-2-specific memory B cell (B_{MEM} – $CD3^-CD19^+CD20^+IgD^-CD38^{int/low}$) frequencies via B-cell bait staining. Frequencies of B_{MEM}-binding Omicron BA.1 and Wuhan full-length S glycoprotein (**a**) or RBD (**b**) for Omicron BA.1-experienced and naïve BNT162b2 double and triple vaccinees are shown. Mean values are indicated. n = number of individuals per group.

```
RBD
Wuhan WT strain
VOD ALPHA
VOC DELTA
VOC OMICRON BA.1
VOC OMICRON BA.4/5

301  CTLKSFTVEKGIYQTSNFRVQPTESIVRFPNITNLCPFGEVFNATRFASV
301  CTLKSFTVEKGIYQTSNFRVQPTESIVRFPNITNLCPFGEVFNATRFASV
301  CTLKSFTVEKGIYQTSNFRVQPTESIVRFPNITNLCPFGEVFNATRFASV
301  CTLKSFTVEKGIYQTSNFRVQPTESIVRFPNITNLCPFDEVFNATRFASV
301  CTLKSFTVEKGIYQTSNFRVQPTESIVRFPNITNLCPFDEVFNATRFASV

351  YAWNRKRISNCVADYSVLYNSASFSTFKCYGVSPTKLNDLCFTNVYADSF
351  YAWNRKRISNCVADYSVLYNSASFSTFKCYGVSPTKLNDLCFTNVYADSF
351  YAWNRKRISNCVADYSVLYNSASFSTFKCYGVSPTKLNDLCFTNVYADSF
351  YAWNRKRISNCVADYSVLYNLAPFFTFKCYGVSPTKLNDLCFTNVYADSF
351  YAWNRKRISNCVADYSVLYNFAPFFAFKCYGVSPTKLNDLCFTNVYADSF

401  VIRGDEVRQIAPGQTGKIADYNYKLPDDFTGCVIAWNSNNLDSKVGGNYN
401  VIRGDEVRQIAPGQTGKIADYNYKLPDDFTGCVIAWNSNNLDSKVGGNYN
401  VIRGDEVRQIAPGQTGKIADYNYKLPDDFTGCVIAWNSNNLDSKVGGNYN
401  VIRGDEVRQIAPGQTGNIADYNYKLPDDFTGCVIAWNSNKLDSKVSGNYN
401  VIRGNEVSQIAPGQTGNIANYNYKLPDDFTGCVIAWNSNKLDSKVGGNYN

451  YLYRLFRKSNLKPFERDISTEIYQAGSTPCNGVEGFNCYFPLQSYGFQPT
451  YLYRLFRKSNLKPFERDISTEIYQAGSTPCNGVEGFNCYFPLQSYGFQPT
451  YRYRLFRKSNLKPFERDISTEIYQAGSKPCNGVEGFNCYFPLQSYGFQPT
451  YLYRLFRKSNLKPFERDISTEIYQAGNKPCNGVAGFNCYFPLRSYSFRPT
451  YRYRLFRKSNLKPFERDISTEIYQAGNKPCNGVAGVNCYFPLQSYGFRPT

501  NGVGYQPYRVVVLSFELLHAPATVCGPKKSTNLVKNKCVNFNFNGLTGTG
501  YGVGYQPYRVVVLSFELLHAPATVCGPKKSTNLVKNKCVNFNFNGLTGTG
501  NGVGYQPYRVVVLSFELLHAPATVCGPKKSTNLVKNKCVNFNFNGLTGTG
501  YGVGHQPYRVVVLSFELLHAPATVCGPKKSTNLVKNKCVNFNFNGLKGTG
501  YGVGHQPYRVVVLSFELLHAPATVCGPKKSTNLVKNKCVNFNFNGLTGTG
```

Fig. S6. Sequences of Wuhan and variants RBDs.

Variant-specific amino acid alterations are indicated in bold red font, with the original Wuhan amino acid highlighted in bold blue font.

Fig. S7

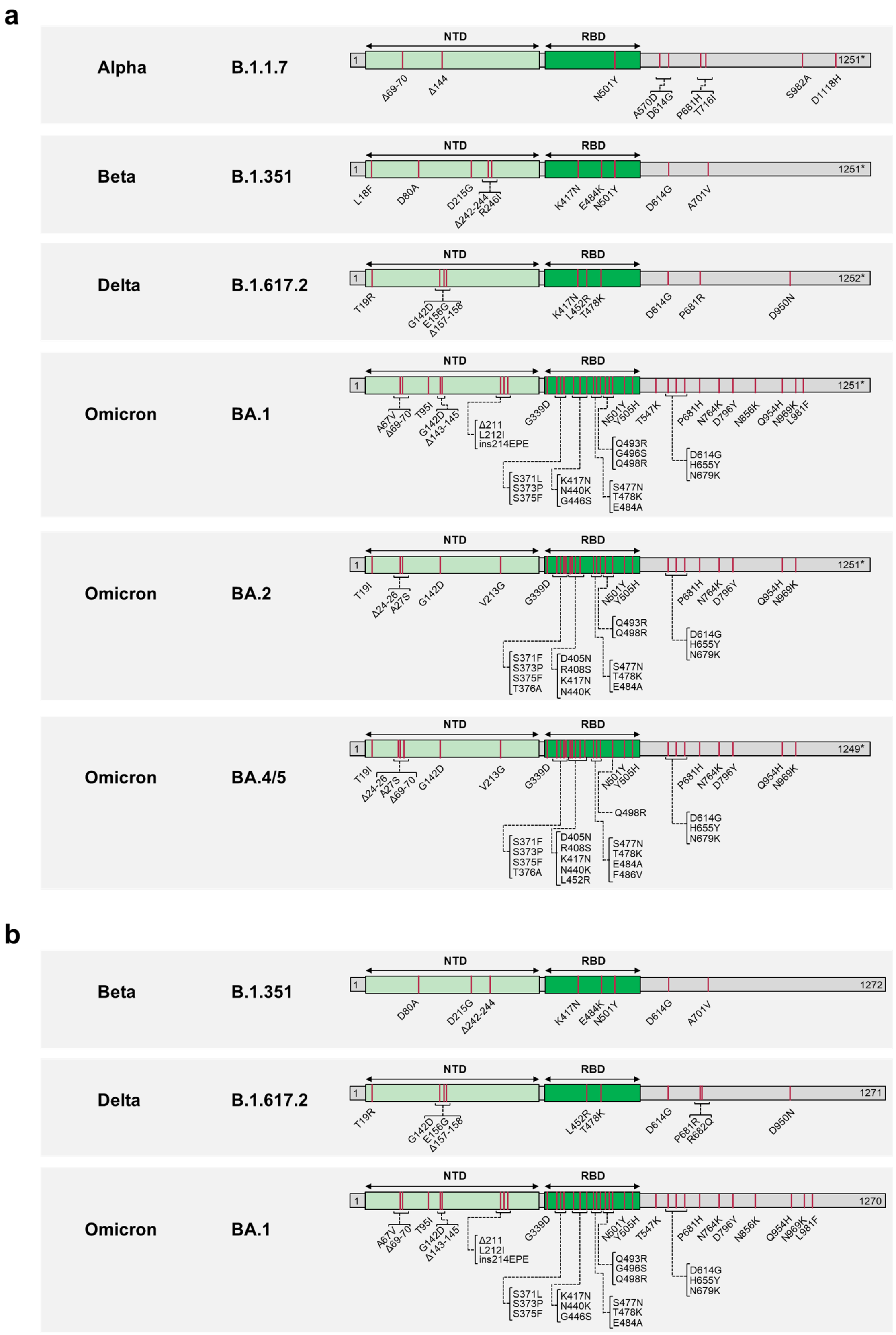
a
Alpha
B.1.1.7
NTD
RBD
1
1251*
Δ69-70
Δ144
N501Y
A570D
D614G
P681H
T716I
S982A
D1118H
Beta
B.1.351
NTD
RBD
1
1251*
L18F
D80A
D215G
Δ242-244
R246I
K417N
E484K
N501Y
D614G
A701V
Delta
B.1.617.2
NTD
RBD
1
1252*
T19R
G142D
E156G
Δ157-158
K417N
L452R
T478K
D614G
P681R
D950N
Omicron
BA.1
NTD
RBD
1
1251*
A67V
Δ69-70
T95I
G142D
Δ143-145
Δ211
L212I
ins214EPE
G339D
S371L
S373P
S375F
K417N
N440K
G446S
S477N
T478K
E484A
Q493R
G496S
Q498R
N501Y
Y505H
T547K
D614G
H655Y
N679K
P681H
N764K
D796Y
N856K
Q954H
N969K
L981F
Omicron
BA.2
NTD
RBD
1
1251*
T19I
Δ24-26
A27S
G142D
V213G
G339D
S371F
S373P
S375F
T376A
D405N
R408S
K417N
N440K
S477N
T478K
E484A
Q493R
Q498R
N501Y
Y505H
D614G
H655Y
N679K
P681H
N764K
D796Y
Q954H
N969K
Omicron
BA.4/5
NTD
RBD
1
1249*
T19I
Δ24-26
A27S
Δ69-70
G142D
V213G
G339D
S371F
S373P
S375F
T376A
D405N
R408S
K417N
N440K
L452R
S477N
T478K
E484A
F486V
Q498R
N501Y
Y505H
D614G
H655Y
N679K
P681H
N764K
D796Y
Q954H
N969K
b
Beta
B.1.351
NTD
RBD
1
1272
D80A
D215G
Δ242-244
K417N
E484K
N501Y
D614G
A701V
Delta
B.1.617.2
NTD
RBD
1
1271
T19R
G142D
E156G
Δ157-158
L452R
T478K
D614G
P681R
R682Q
D950N
Omicron
BA.1
NTD
RBD
1
1270
A67V
Δ69-70
T95I
G142D
Δ143-145
Δ211
L212I
ins214EPE
G339D
S371L
S373P
S375F
K417N
N440K
G446S
S477N
T478K
E484A
Q493R
G496S
Q498R
N501Y
Y505H
T547K
D614G
H655Y
N679K
P681H
N764K
D796Y
N856K
Q954H
N969K
L981F

Fig. S7. Characteristics of SARS-CoV-2 S glycoproteins used in the assays based on (a) VSV-SARS-CoV-2 variant pseudoviruses and (b) live authentic SARS-CoV-2.

The sequence of the Wuhan-Hu-1 isolate SARS-CoV-2 S glycoprotein (GenBank: QHD43416.1) was used as reference. Amino acid positions, amino acid descriptions (one letter code) and kind of mutations (substitutions, deletions, insertions) are indicated. NTD, N-terminal domain; RBD, receptor-binding domain; Δ, deletion; ins, insertion; *, cytoplasmic domain truncated for the C-terminal 19 amino acids.

Table S1. BNT162b2 vaccinated individuals analyzed for neutralizing antibody responses.

Characteristic	BNT162b2[2] (n=20)	BNT162b2[3] (n=19)	BNT162b2[2] + Omi (n=8)	BNT162b2[3] + Omi (n=10)
Sex, n (%)				
Male	10 (50)	10 (53)	3 (38)	7 (70)
Female	10 (50)	9 (47)	5 (62)	3 (30)
Age, median (range)	52 (23-68)	38 (23-54)	39 (27-60)°	32 (23-60)°
Age group at vaccination, n (%)				
18-55 yrs	12 (60)	19 (100)	6 (75)	9 (90)
56-85 yrs	8 (40)	0 (0)	2 (25)	1 (10)
Baseline SARS-CoV-2 status, n (%)				
Positive	0 (0)	0 (0)	8 (100)†	10 (100)†
Negative	20 (100)*	19 (100)[#]	0 (0)	0 (0)
Unknown	0 (0)	0 (0)	0 (0)	0 (0)
Interval, median (range)				
Days between D1/D2	20 (19-21)	‡	40 (21-49)	38 (20-92)
Days until serum draw after D2	22 (20-23)	N/A	N/A	n/a
Days between D2/D3	N/A	202 (181-266)	N/A	192 (159-243)
Days until serum draw after D3	N/A	28 (26-30)	N/A	N/A
Days between last dose/infection	N/A	N/A	153 (142-182)	31 (3-112)
Days until serum draw after infection	N/A	N/A	46 (41-54)	44 (25-55)

N/A: not applicable; n/a, not available; D, Dose; Yrs, Years; n, Number.
*, Negative SARS-CoV-2 PCR test at the time of enrollment
[#], No evidence of prior SARS-CoV-2 infection (based on COVID-19 symptoms/signs and SARS-CoV-2 PCR test)
°, Age is estimated based on the indicated year of birth in Table S1
‡, Participants received the primary 2-dose series of BNT162b2 vaccine as part of a governmental vaccination program and the interval between doses was not recorded
†, Omicron BA.1 infection confirmed at time of recruitment to the research study

Table S2. BNT162b2 vaccinated individuals analyzed for frequencies of full S glycoprotein/RBD-specific B_{MEM} cells.

Characteristic	BNT162b2² (n=7)	BNT162b2³ (n=5)	BNT162b2² + Omi (n=7)	BNT162b2³ + Omi (n=5)
**Overlap with pVNT/ VNT cohorts (n)**	**n=4**	**n=0**	**n=7**	**n=5**
Sex, n (%)				
Male	4 (57)	4 (80)	3 (43)	4 (80)
Female	3 (43)	1 (20)	4 (57)	1 (20)
Age, median (range)	51 (23-80)	26 (20-69)	42 (29-60)°	29 (23-55)°
Age group at vaccination, n (%)				
18-55 yrs	5 (71)	3 (60)	5 (71)	5 (100)
56-85 yrs	2 (29)	2 (40)	2 (29)	0 (0)
Baseline SARS-CoV-2 status, n (%)				
Positive	0 (0)	0 (0)	7 (100)†	5 (100)†
Negative	7 (100)*	5 (100)#	0 (0)	0 (0)
Unknown	0 (0)	0 (0)	0 (0)	0 (0)
Interval, median (range)				
Days between D1/D2	21 (19-23)	21 (19-23)	42 (21-49)	36 (28-42)
Days until early blood draw after D2	22 (19-23)	N/A	N/A	N/A
Days until late blood draw after D2	162 (160-167)	N/A	N/A	N/A
Days between D2/D3	N/A	251 (180-276)	N/A	189 (159-255)
Days until blood draw after D3	N/A	84 (81-86)	N/A	N/A
Days between last dose/infection	N/A	N/A	155 (142-182)	10 (3-27)
Days until blood draw after infection	N/A	N/A	46 (41-54)	44 (43-47)

N/A, not applicable; D, Dose; Yrs, Years; n, Number.

*, Negative SARS-CoV-2 PCR test at the time of enrollment
#, No evidence of prior SARS-CoV-2 infection (based on COVID-19 symptoms/signs and SARS-CoV-2 PCR test)
°, Age is estimated based on the indicated year of birth in Table S1
†, Omicron BA.1 infection confirmed at time of recruitment to the research study

Table S3. Double and triple BNT162b2-vaccinated individuals with Omicron breakthrough infection.

Participant ID	YOB	Sex	Vaccination BNT162b2	Omicron subtype	Dose 1-2 interval	Dose 2-3 interval	Positive test after last vaccination	Blood draw after positive test	Severity (WHO grade)
**1**	1991	f	2 doses	n/a	42	N/A	170	46	1-2
**2**	1987	m	2 doses	BA.1	21	N/A	143	51	1-2
**3**	1995	f	2 doses	BA.1	33	N/A	151	52	1-2
**4**	1977	m	2 doses	BA.1	42	N/A	142	43	1-2
**5**	1966	f	2 doses	BA.1	38	N/A	169	54	1-2
**6**	1993	f	2 doses	n/a	42	N/A	182	41	1-2
**7**	1962	f	2 doses	n/a	35	N/A	155	41	1-2
**8**	1980	m	2 doses	n/a	49	N/A	148	46	1-2
**9**	1990	f	3 doses	n/a	24	243	64	55	1-2
**10**	1990	m	3 doses	n/a	20	233	66	53	1-2
**11**	1994	m	3 doses	n/a	35	213	10	47	1-2
**12**	1993	f	3 doses	BA.1	36	189	3	46	1-2
**13**	1999	m	3 doses	n/a	42	159	27	44	1-2
**14**	1991	m	3 doses	n/a	42	166	20	43	1-2
**15**	1969	m	3 doses	n/a	39	194	22	25	1-2
**16**	1972	f	3 doses	n/a	92	169	35	28	1-2
**17**	1972	m	3 doses	n/a	42	169	44	31	1-2
**18**	1962	m	3 doses	n/a	26	236	112	43	1-2
						**Median**	65	45	

YOB, year of birth; m, male; f, female; n/a, not available; N/A, not applicable;

.

Table S4. pVN_{50} values of sera collected from Omicron-naïve double BNT162b2-vaccinated individuals.

Clinical trial	Participant ID	pVN50							
		Wuhan	Alpha	Beta	Delta	Omicron BA.1	Omicron BA.2	Omicron BA.4/5	SARS-CoV-1
BNT162-01	19	160	40	10	40	5	5	5	5
	20	320	n/a	n/a	n/a	5	n/a	n/a	n/a
	21	80	n/a	n/a	80	5	n/a	5	n/a
	22	160	160	40	80	10	20	20	5
	23	320	160	320	320	10	40	80	5
	24	160	80	20	80	5	10	5	5
	25	160	80	20	80	5	10	5	10
	26	320	160	80	160	20	80	20	20
	27	160	80	40	40	5	40	10	5
	28	160	80	20	80	10	20	5	5
	29	160	40	10	80	5	5	5	5
	30	160	80	10	40	5	10	5	5
	31	160	80	160	80	5	20	40	5
	32	80	40	10	40	5	5	5	5
	33	160	160	20	80	5	20	10	5
	34	640	320	40	160	10	20	5	5
	35	160	320	80	160	10	20	5	5
	36	160	80	20	80	5	20	10	5
	37	20	20	5	5	5	5	5	5
	38	160	80	10	80	5	5	5	5

n/a, not available due to lack of serum.

Table S5. pVN_{50} values of sera collected from Omicron-naïve triple BNT162b2-vaccinated individuals.

Clinical trial	Participant ID	pVN50							
		Wuhan	Alpha	Beta	Delta	Omicron BA.1	Omicron BA.2	Omicron BA.4/5	SARS-CoV-1
BNT162-17	39	160	320	160	160	80	160	40	5
	40	320	640	320	640	320	320	320	10
	41	640	640	320	320	160	320	40	40
	42	5120	5120	1280	2560	1280	1280	640	40
	43	320	640	160	320	160	160	40	20
	44	640	640	80	640	320	160	40	20
	45	320	640	160	320	160	160	40	10
	46	320	640	320	320	160	160	80	10
	47	320	640	320	320	160	160	80	20
	48	160	320	80	160	40	80	40	20
	49	320	1280	160	320	160	60	80	20
	50	1280	5120	640	1280	640	640	320	80
	51	40	40	20	20	5	40	5	5
	52	320	640	320	320	80	160	40	20
	53	160	320	160	320	80	160	40	20
	54	320	640	320	320	320	320	160	20
	55	640	640	320	320	160	320	80	40
	56	2560	5120	640	1280	640	640	320	80
	57	320	640	160	640	160	320	80	20

Table S6. pVN_{50} values of sera collected from double and triple BNT162b2-vaccinated individuals after confirmed Omicron BA.1 breakthrough infection.

Participant ID	pVN_{50}							
	Wuhan	Alpha	Beta	Delta	Omicron BA.1	Omicron BA.2	Omicron BA.4/5	SARS-CoV-1
1*	960	1920	960	960	960	480	160	120
2*	960	1920	960	960	1920	480	160	120
3*	960	1920	960	480	960	960	160	120
4*	480	480	480	240	480	480	40	120
5*	240	480	240	120	480	120	40	5
6*	1920	1920	1920	1920	960	960	640	40
7*	960	1920	960	960	960	960	160	40
8*	480	960	480	480	960	480	160	20
9#	1920	3840	1920	1920	1920	960	320	120
10#	960	1920	960	480	480	480	160	120
11#	3840	3840	3840	3840	1920	1920	640	960
12#	960	1920	960	960	960	960	160	120
13#	480	1920	960	480	480	480	40	20
14#	1920	1920	960	960	960	1920	320	40
15#	960	1920	960	960	1920	480	80	80
16#	960	1920	480	960	480	480	80	5
17#	480	960	480	480	480	480	80	60
18#	1920	3840	3840	3840	3840	1920	2560	120

*, participant received two doses of BNT162b2 prior to Omicron BA.1 infection
#, participant received three doses of BNT162b2 prior to Omicron BA.1 infection

Table S7. VN_{50} values of sera collected from Omicron-naïve double BNT162b2-vaccinated individuals.

Clinical trial	Participant ID	VN_{50}			
		Wuhan	Beta	Delta	Omicron BA.1
BNT162-01	19	226	5	20	5
	20	320	14	80	5
	21	226	5	40	5
	22	905	28	80	7
	23	640	113	226	10
	24	320	14	20	5
	25	453	28	80	14
	26	1810	80	160	28
	27	453	28	40	7
	28	320	14	80	7
	29	226	5	20	5
	30	453	14	28	5
	31	320	28	40	5
	32	160	14	14	5
	33	640	20	80	5
	34	320	20	20	7
	35	320	28	40	7
	36	640	20	56	5
	37	40	5	5	5
	38	320	7	40	5

Table S8. VN_{50} titer values of sera collected from Omicron-naïve triple BNT162b2-vaccinated individuals.

Clinical trial	Participant ID	VN_{50} titer			
		Wuhan	Beta	Delta	Omicron BA.1
BNT162-17	39	226	80	226	40
	40	905	453	320	226
	41	640	226	226	160
	42	3620	1280	1810	453
	43	226	226	160	40
	44	226	80	160	57
	45	640	113	226	80
	46	453	226	453	80
	47	640	226	226	160
	48	160	80	113	28
	49	640	80	320	80
	50	3620	905	1280	453
	51	28	10	10	5
	52	453	113	226	40
	53	226	113	226	57
	54	905	160	453	160
	55	1280	160	226	113
	56	3620	905	1810	1280
	57	905	226	320	80

Table S9. VN_{50} titer values of sera collected from double and triple BNT162b2-vaccinated individuals after confirmed Omicron BA.1 breakthrough infection.

Participant ID	VN_{50} titer			
	Wuhan	Beta	Delta	Omicron BA.1
1*	453	453	453	640
2*	640	905	1280	640
3*	453	640	320	640
4*	160	453	226	320
5*	80	113	160	453
6*	1280	905	1280	640
7*	905	905	640	453
8*	226	320	320	320
9#	453	1280	1280	640
10#	453	640	640	453
11#	1810	1810	2560	1810
12#	453	905	1280	453
13#	640	640	320	453
14#	640	640	905	640
15#	320	640	453	453
16#	905	226	453	226

*, participant received two doses of BNT162b2 prior to Omicron BA.1 infection
#, participant received three doses of BNT162b2 prior to Omicron BA.1 infection

Table S10. Individuals vaccinated with other approved COVID-19 vaccines or mixed regimens after subsequent Omicron breakthrough infection.

Participant ID	YOB	Sex	Vaccination	Omicron subtype	Dose 1-2 interval	Dose 2-3 interval	Positive test after last vaccination	Blood draw after positive test	Severity (WHO grade)
**58**	1960	f	AZ/BNT	n/a	62	N/A	142	46	1-2
**59**	1955	m	AZ/BNT	n/a	68	N/A	135	45	1-2
**60**	1983	m	J&J	n/a	N/A	N/A	161	44	1-2
**61**	1962	m	MOD^{3}	BA.1	42	172	3	35	1-2
**62**	1993	f	MOD^{2}	n/a	42	N/A	169	44	1-2
**63**	1981	m	J&J/BNT	n/a	138	N/A	45	40	1-2
**64**	1989	m	AZ/BNT/MOD	BA.1	68	154	9	43	1-2
**65**	1994	m	MOD^{2}/BNT	n/a	28	252	22	40	1-2
**66**	1990	m	MOD^{2}/BNT	n/a	42	154	13	42	1-2
**67**	1972	m	MOD^{2}/BNT	n/a	28	256	45	31	1-2
						**Median:**	45	43	

OB, year of birth; m, male; f, female; n/a, not available; N/A, not applicable;
AZ, AstraZeneca AZD1222; BNT, BioNTech/Pfizer BNT162b2; J&J, Johnson & Johnson Ad26.COV2.S; MOD, Moderna mRNA-1273; BNT^{4}, BNT162b2 four-dose series; MOD^{2}, mRNA-1273 two-dose series; MOD^{3}, mRNA-1273 three-dose series
.

Table S11. pVN$_{50}$ titer values of sera collected from individuals vaccinated with other approved COVID-19 vaccines or mixed regimens after subsequent Omicron BA.1 breakthrough infection.

Participant ID	pVN$_{50}$ titer							
	Wuhan	Alpha	Beta	Delta	Omicron BA.1	Omicron BA.2	Omicron BA.4/5	SARS-CoV-1
58	1920	3840	1920	960	960	960	640	240
59	1920	3840	960	1920	1920	960	320	10
60	120	480	120	240	120	60	40	60
61	7680	15360	7680	3840	15360	3840	1280	480
62	120	120	30	30	60	60	5	5
63	480	1920	960	960	480	480	320	120
64	480	1920	960	480	240	480	320	60
65	480	240	60	240	60	120	40	30
66	1920	1920	960	1920	960	960	640	60
67	3840	15360	7680	7680	3840	3840	2560	120

Table S12. Frequencies of S glycoprotein/RBD-specific B_{MEM} cells for all individuals analyzed.

Study	Participant ID	Wuhan Spike⁺ BMEMs	Alpha Spike⁺ BMEMs	Delta Spike⁺ BMEMs	Omicron BA.1 Spike⁺ BMEMs	Wuhan RBD⁺ BMEMs	Alpha RBD⁺ BMEMs	Delta RBD⁺ BMEMs	Omicron BA.1 RBD⁺ BMEMs
Investigator Study	1*	3.87	3.16	3.16	3.79	0.49	0.52	0.50	0.47
	2*	1.09	0.88	0.94	0.98	0.62	0.66	0.46	0.47
	4*	0.94	0.81	0.72	1.00	0.23	0.33	0.20	0.21
	5*	0.71	0.54	0.63	0.78	1.96	1.96	1.38	1.49
	6*	2.15	1.83	1.91	2.09	1.15	1.17	1.10	0.89
	7*	2.57	2.09	2.25	2.53	2.06	2.13	1.86	1.59
	8*	0.45	0.33	0.34	0.62	0.21	0.26	0.21	0.21
	11#	0.73	0.73	0.93	0.90	0.37	0.29	0.23	0.34
	12#	1.38	1.34	1.86	1.87	1.12	0.93	0.72	1.06
	13#	1.67	1.65	2.15	2.05	1.12	0.94	0.68	1.06
	14#	1.35	1.33	1.68	1.62	0.94	0.75	0.72	0.91
	15#	2.75	2.77	3.40	3.11	2.53	2.22	1.68	2.41
	16#	0.58	0.61	0.82	0.73	0.50	0.44	0.29	0.42
BNT162-01 Early	35	0.28	0.18	0.19	0.27	0.11	0.12	0.13	0.05
	38	0.33	0.18	0.25	0.29	0.08	0.09	0.09	0.03
	41	0.19	0.13	0.14	0.18	0.05	0.08	0.07	0.04
	47	0.31	0.20	0.16	0.31	0.07	0.10	0.08	0.02
	69	0.19	0.16	0.16	0.18	0.07	0.11	0.08	0.04
	70	0.59	0.39	0.45	0.44	0.23	0.26	0.19	0.15
	71	0.52	0.37	0.46	0.46	0.16	0.23	0.17	0.07
BNT162-01 Late	38	0.86	0.87	1.17	1.27	0.46	0.35	0.23	0.39
	41	0.37	0.39	0.67	0.60	0.23	0.18	0.12	0.18
	69	0.31	0.36	0.55	0.47	0.18	0.13	0.06	0.09
	70	1.97	2.05	2.67	2.65	0.68	0.55	0.44	0.66
	71	1.14	1.27	1.71	1.64	0.43	0.28	0.17	0.31
BNT162-14	72	0.96	0.75	0.75	0.89	0.30	0.35	0.27	0.15
	73	0.62	0.44	0.45	0.62	0.23	0.26	0.28	0.16
	74	1.92	1.35	1.55	1.48	0.62	0.62	0.59	0.50
	75	1.36	1.06	0.94	1.25	0.41	0.47	0.40	0.25
	76	2.01	1.58	1.58	1.86	0.64	0.68	0.62	0.49

*, subject received two doses of BNT162b2 prior to Omicron BA.1 infection
#, subject received three doses of BNT162b3 prior to Omicron BA.1 infection

Table S13. Statistical analysis corresponding to Figure 4 c-g; nonparametric Friedman test with Dunn's multiple comparisons correction

Figure	Group tested	p value (Shared vs. Wuhan)	p value (Shared vs. Omi BA.1)
**4c**	BNT162b2^2 early (n=7)	>0.9999	0.1647
	BNT162b2^2 late (n=5)	0.0089	0.1156
	BNT162b2^2 + Omi (n=7)	0.0066	0.0151
	BNT162b2^3 (n=5)	0.164	0.0053
	BNT162b2^3 + Omi (n=6)	0.003	0.0866
**4d**	BNT162b2^2 early (n=7)	0.8454	0.0027
	BNT162b2^2 late (n=5)	0.2277	0.0031
	BNT162b2^2 + Omi (n=7)	0.1227	0.0004
	BNT162b2^3 (n=5)	>0.9999	0.0187
	BNT162b2^3 + Omi (n=6)	0.1665	0.0011
**Figure**	**Group tested**	**p value (All 4 +ve vs. Only Wuhan)**	**p value (All 4 +ve vs. Only Omi BA.1)**
**4f**	BNT162b2^2 early (n=7)	0.3629	>0.9999
	BNT162b2^2 late (n=5)	0.0031	0.2277
	BNT162b2^2 + Omi (n=7)	0.0004	0.1227
	BNT162b2^3 (n=5)	0.0089	0.1156
	BNT162b2^3 + Omi (n=6)	0.0011	0.1665
**4g**	BNT162b2^2 early (n=7)	>0.9999	0.0066
	BNT162b2^2 late (n=5)	0.0797	0.0144
	BNT162b2^2 + Omi (n=7)	0.0066	0.0151
	BNT162b2^3 (n=5)	0.2277	0.0031
	BNT162b2^3 + Omi (n=6)	0.0418	0.0078
