# Supplementary material for: Omicron BA.1 breakthrough infection drives cross-variant neutralization and memory B cell formation against conserved epitopes
Source: Sci Immunol. 2022 Jun 2:eabq2427. doi: 10.1126/sciimmunol.abq2427 (PMC9162083; doi:10.1126/sciimmunol.abq2427)
Supplement: Supplementary file 2 — MDAR Reproducibility Checklist [file sciimmunol.abq2427_reproducibility_checklist.pdf]

Materials Design Analysis Reporting (MDAR) Checklist for Authors

The MDAR framework establishes a minimum set of requirements in transparent reporting applicable to studies in the life sciences (see Statement of Task: doi:10.31222/osf.io/9sm4x.). The MDAR checklist is a tool for authors, editors, and others seeking to adopt the MDAR framework for transparent reporting in manuscripts and other outputs. Please refer to the MDAR Elaboration Document for additional context for the MDAR framework.

For all that apply, please note where in the manuscript the required information is provided.

Materials:

Newly created materials	indicate where provided: page no/section/legend)	n/a
The manuscript includes a dedicated "materials availability statement" providing transparent disclosure about availability of newly created materials including details on how materials can be accessed and describing any restrictions on access.	N/A: all materials were either purchased and are freely available, or was clinical trial	

Antibodies	indicate where provided: page no/section/legend)	n/a
For commercial reagents, provide supplier name, catalogue number and RRID, if available.	Materials and Methods – VSV-SARS-CoV-2 S variant pseudovirus generation / Detection and characterization of SARS-CoV-2-specific B cells with flow cytometry (page 21, 24-25, Materials and Methods)<br><br>anti-VSV-G antibody (clone 8G5F11, Kerafast Inc.)<br>anti-CD3 (clone UCHT1, BD Biosciences)<br>anti-CD4 (clone SK3, BD Biosciences)<br>anti-CD185 (clone RF8B2, BioLegend)<br>anti-CD279 (clone EH12.1, BD Biosciences)<br>anti-CD278 (clone C398.4A, BioLegend)<br>anti-CD19 (clone SJ25C1, BD Biosciences)<br>anti-CD20 (clone 2H7, BD Biosciences)<br>anti-CD38 (clone HIT2, BD Biosciences)<br>anti-CD11c (clone S-HCL-3, BD Biosciences)<br>anti-CD138 (clone MI15, BD Biosciences)<br>anti-IgG (clone G18-145, BD Biosciences)<br>anti-IgM (clone G20-127, BD Biosciences)<br>anti-IgD (clone IA6-2, BD Biosciences)<br>anti-CD14 (clone MφP9, BD Biosciences)<br>anti-CD16 (clone 3G8, BD Biosciences)	(

DNA and RNA sequences	indicate where provided: page no/section/legend)	n/a
**Short novel DNA or RNA including primers, probes:** Sequences should be included or deposited in a public repository.		X

Cell materials	indicate where provided: page no/section/legend	n/a
**Cell lines:** Provide species information, strain. Provide accession number in repository **OR** supplier name, catalog number, clone number, **OR** RRID.	Materials and Methods – VSV-SARS-CoV-2 S variant pseudovirus generation / Live SARS-CoV-2 neutralization assay (pages 21 and 23, Materials and Methods):<br><br>HEK293T/17 (ATCC® CRL-11268™)<br>VERO 76 (ATCC® CRL-1587™)<br>Vero E6 (ATCC® CRL-1586™)	
**Primary cultures:** Provide species, strain, sex of origin, genetic modification status.		X

Experimental animals	indicate where provided: page no/section/legend)	n/a
**Laboratory animals or Model organisms:** Provide species, strain, sex, age, genetic modification status. Provide accession number in repository **OR** supplier name, catalog number, clone number, **OR** RRID.		X
**Animal observed in or captured from the field:** Provide species, sex, and age where possible.		X

Plants and microbes	indicate where provided: page no/section/legend)	n/a
**Plants:** provide species and strain, ecotype and cultivar where relevant, unique accession number if available, and source (including location for collected wild specimens).		X
**Microbes:** provide species and strain, unique accession number if available, and source.		X

Human research participants	indicate where provided: page no/section/legend) or state if these demographics were not collected	n/a
If collected and within the bounds of privacy constraints report on age, sex and gender or ethnicity for all study participants.	Materials and Methods – Study design and recruitment of participants and sample collection (pages 18 - 20):<br><br>Demographic and clinical information for all participants as well as sampling timepoints are provided in Tables S1-S3 and S10, and Fig. 1.	

Design:

Study protocol	indicate where provided: page no/section/legend)	n/a
If study protocol has been pre-registered, provide DOI. For clinical trials, provide the trial registration number **OR** cite DOI.	Trial NCT numbers are listed in M&M	

Laboratory protocol	indicate where provided: page no/section/legend)	n/a
Provide DOI **OR** other citation details if detailed step-by-step protocols are available.		X

Experimental study design (statistics details)		
**For in vivo studies:** State whether and how the following have been done	**indicate where provided: page no/section/legend. If it could have been done, but was not, write not done**	**n/a**
Sample size determination		X
Randomisation		X
Blinding		X
Inclusion/exclusion criteria		X

Sample definition and in-laboratory replication	indicate where provided: page no/section/legend	n/a
State number of times the experiment was replicated in laboratory.		
Define whether data describe technical or biological replicates.	Materials and Methods – Pages 20 - 25<br><br>Pseudovirus neutralization assay: n=2 technical replicates per serum per pseudovirus<br><br>Live SARS-CoV-2 neutralization assay: n=2 technical replicates per serum per virus<br><br>Detection and characterization of SARS-CoV-2-specific B cells with flow cytometry: single samples per participant	

Ethics	indicate where provided: page no/section/legend	n/a
**Studies involving human participants:** State details of authority granting ethics approval (IRB or equivalent committee(s), provide reference number for approval.		X
**Studies involving experimental animals:** State details of authority granting ethics approval (IRB or equivalent committee(s), provide reference number for approval.		X
**Studies involving specimen and field samples:** State if relevant permits obtained, provide details of authority approving study; if none were required, explain why.	Materials and Methods – Recruitment of participants and sample collection (pages 19- 20):<br><br>Individuals from the SARS-CoV-2 Omicron-naïve BNT162b2 double-vaccinated (BNT162b22) and triple-vaccinated (BNT162b23) cohorts provided informed consent as part of their participation in a clinical trial (the Phase 1/2 trial BNT162-01 [NCT04380701], the Phase 2 rollover trial BNT162-14 [NCT04949490], or as	

	part of the BNT162-17 [NCT05004181] trial). Participants from the SARS-CoV-2 Omicron convalescent double- and triple vaccinated cohorts (BNT162b22 + Omi and BNT162b23 + Omi cohorts, respectively) and individuals vaccinated with other approved COVID-19 vaccines or mixed regimens with subsequent Omicron breakthrough infection were recruited from University Hospital, Goethe University Frankfurt as part of a research program that recruited patients that had experienced Omicron breakthrough infection following vaccination for COVID-19, to provide blood samples and clinical data for research. The study protocol for this research program was approved by the Ethics Board of the University Hospital, Goethe University Frankfurt (No. 2021-560).	

Dual Use Research of Concern (DURC)	indicate where provided: page no/section/legend	n/a
If study is subject to dual use research of concern regulations, state the authority granting approval and reference number for the regulatory approval.		X

Analysis:

Attrition	indicate where provided: page no/section/legend	n/a
Describe whether exclusion criteria were preestablished. Report if sample or data points were omitted from analysis. If yes report if this was due to attrition or intentional exclusion and provide justification.	Materials and Methods – Study design (page 19)<br><br>All participants had no documented history of SARS-CoV-2 infection prior to vaccination.	X

Statistics	indicate where provided: page no/section/legend	n/a
Describe statistical tests used and justify choice of tests.	Materials and Methods – Statistical analysis (pages 25 - 26):<br><br>The statistical method of aggregation used for the analysis of antibody titers is the geometric mean and for the ratio of SARS-CoV-2 VOC titer and Wuhan titer the geometric mean and the corresponding 95% confidence interval. The use of the geometric mean accounts for the non-normal distribution of antibody titers, which span several orders of magnitude. The Friedman test with Dunn's correction for multiple comparisons was used to conduct pairwise signed-rank tests of group geometric mean neutralizing antibody titers with a common control group. Flow cytometric frequencies were analyzed with and tables were exported from FlowJo software (Version 10.7.1.). Statistical analysis of cumulative memory B cell frequencies was the mean and standard errors of the mean (SEM). Statistical significance was tested for using the non-parametric Friedman test with Dunn's multiple comparisons correction. All statistical analyses were performed using GraphPad Prism software version 9.	

Data availability	indicate where provided: page no/section/legend	n/a
For newly created and reused datasets, the manuscript includes a data availability statement that provides details for access or notes restrictions on access.	Section – Data and materials availability, page 42	
If newly created datasets are publicly available, provide accession number in repository **OR** DOI **OR** URL and licensing details where available.		X
If reused data is publicly available provide accession number in repository **OR** DOI **OR** URL, **OR** citation.		X

Code availability	indicate where provided: page no/section/legend	n/a
For all newly generated custom computer code/software/mathematical algorithm or re-used code essential for replicating the main findings of the study, the manuscript includes a data availability statement that provides details for access or notes restrictions.		X
If newly generated code is publicly available, provide accession number in repository, **OR** DOI **OR** URL and licensing details where available. State any restrictions on code availability or accessibility.		X

If reused code is publicly available provide accession number in repository **OR** DOI **OR** URL, **OR** citation.		X

Reporting

MDAR framework recommends adoption of discipline-specific guidelines, established and endorsed through community initiatives. Journals have their own policy about requiring specific guidelines and recommendations to complement MDAR.

Adherence to community standards	indicate where provided: page no/section/legend	n/a
State if relevant guidelines (e.g., ICMJE, MIBBI, ARRIVE) have been followed, and whether a checklist (e.g., CONSORT, PRISMA, ARRIVE) is provided with the manuscript.		X
